# Supplementary material for: Barriers to implementing contingency management at a methadone treatment clinic: A qualitative study at a tertiary hospital in Tanzania
Source: PLoS One. 2025 Mar 3;20(3):e0314168. doi: 10.1371/journal.pone.0314168 (PMC11875383; doi:10.1371/journal.pone.0314168)
Supplement: S1 File — (DOCX) [file pone.0314168.s001.docx]

COREQ (Consolidated criteria for Reporting Qualitative research) Checklist

Barriers to implementing Contingency management at a methadone treatment clinic. A qualitative study at a tertiary Hospital in Tanzania.

| **Topic** | **Item No.** | **Guide Questions/Description** | **Reported on**  **Page No.** |
| --- | --- | --- | --- |
| **Domain 1: Research team**  **and reﬂexivity** | | | |
| *Personal characteristics* | | | |
| Interviewer/facilitator | 1 | Which author/s conducted the interview or focus group? | BEM- described in page 4 |
| Credentials | 2 | What were the researcher’s credentials? E.g. PhD, MD | MD |
| Occupation | 3 | What was their occupation at the time of the study? | Health care providers  Page 1 |
| Gender | 4 | Was the researcher male or female? | Male |
| Experience and training | 5 | What experience or training did the researcher have? | Training in Research methods |
| *Relationship with*  *participants* | | | |
| Relationship established | 6 | Was a relationship established prior to study commencement? | Yes |
| Participant knowledge of  the interviewer | 7 | What did the participants know about the researcher? e.g. personal  goals, reasons for doing the research |  |
|  |  |  | Reasons for the research |
|  |  |  |  |
| Interviewer characteristics | 8 | What characteristics were reported about the inter viewer/facilitator?  e.g. Bias, assumptions, reasons and interests in the research topic |  |
|  |  |  | Interests in the research topic |
|  |  |  |  |
| **Domain 2: Study design** | | | |
| *Theoretical framework* | | | |
| Methodological orientation and Theory | 9 | What methodological orientation was stated to underpin the study? e.g. grounded theory, discourse analysis, ethnography, phenomenology,  content analysis |  |
|  |  |  | CFIR  Page 3 & 4 |
|  |  |  |  |
| *Participant selection* | | | |
| Sampling | 10 | How were participants selected? e.g. purposive, convenience,  consecutive, snowball |  |
|  |  |  | Purposive |
|  |  |  |  |
| Method of approach | 11 | How were participants approached? e.g. face-to-face, telephone, mail,  email |  |
|  |  |  | Face to face  Page 4 |
|  |  |  |  |
| Sample size | 12 | How many participants were in the study? | 15 |
| Non-participation | 13 | How many people refused to participate or dropped out? Reasons? | None |
| *Setting* | | | |
| Setting of data collection | 14 | Where was the data collected? e.g. home, clinic, workplace | Clinic |
| Presence of non-  participants | 15 | Was anyone else present besides the participants and researchers? |  |
|  |  |  | No |
|  |  |  |  |
| Description of sample | 16 | What are the important characteristics of the sample? e.g. demographic  data, date |  |
|  |  |  | Service users, clinic providers and leaders involved in managing methadone services  Page 4 |
|  |  |  |  |
| *Data collection* | | | |
| Interview guide | 17 | Were questions, prompts, guides provided by the authors? Was it pilot  tested? | Clarifications of questions when needed |
|  |  |  |  |
| Repeat interviews | 18 | Were repeat inter views carried out? If yes, how many? | No |
| Audio/visual recording | 19 | Did the research use audio or visual recording to collect the data? | Audio recordings |
| Field notes | 20 | Were field notes made during and/or after the interview or focus group? |  |
| Duration | 21 | What was the duration of the inter views or focus group? | 15-20 minutes |
| Data saturation | 22 | Was data saturation discussed? | Yes  Pg 4 |
| Transcripts returned | 23 | Were transcripts returned to participants for comment and/or | No |

| **Topic** | **Item No.** | **Guide Questions/Description** | **Reported on**  **Page No.** |
| --- | --- | --- | --- |
|  |  | Correction? |  |
| **Domain 3: analysis and**  **ﬁndings** | | | |
| *Data analysis* | | | |
| Number of data coders | 24 | How many data coders coded the data? | Two  Page 4 |
| Description of the coding  tree | 25 | Did authors provide a description of the coding tree? |  |
|  |  |  | Yes |
| Derivation of themes | 26 | Were themes identified in advance or derived from the data? | From the data |
| Software | 27 | What software, if applicable, was used to manage the data? | Nvivo |
| Participant checking | 28 | Did participants provide feedback on the findings? |  |
| *Reporting* | | | |
| Quotations presented | 29 | Were participant quotations presented to illustrate the themes/findings?  Was each quotation identified? e.g. participant number |  |
|  |  |  | Yes  Page 6-9 |
|  |  |  |  |
| Data and findings consistent | 30 | Was there consistency between the data presented and the findings? | Yes |
| Clarity of major themes | 31 | Were major themes clearly presented in the findings? | Yes  Page 6-9 |
| Clarity of minor themes | 32 | Is there a description of diverse cases or discussion of minor themes? | NA |

Developed from: Tong A, Sainsbury P, Craig J. Consolidated criteria for reporting qualitative research (COREQ): a 32-item checklist for interviews and focus groups. *International Journal for Quality in Health Care*. 2007. Volume 19, Number 6: pp. 349 – 357
