# Supplementary material for: Barriers to implementing contingency management at a methadone treatment clinic: A qualitative study at a tertiary hospital in Tanzania
Source: PLoS One. 2025 Mar 3;20(3):e0314168. doi: 10.1371/journal.pone.0314168 (PMC11875383; doi:10.1371/journal.pone.0314168)
Supplement: S2 File — (DOCX) [file pone.0314168.s002.docx]

**Qualitative interview guide for Patients, Hospital administrators and health care providers**

*Assessing barriers on implementation of contingency management for methadone users at Mbeya Zonal Referral Hospital, January – December 2020.*

**GENERAL INFORMATION**

1. Can you tell me your what you know about methadone treatment services ?
2. In your experience of providing/ receiving services at the methadone clinic, what has been the successes and challenges ?

**CONTIGENCY MANAGEMENT-IMPLEMENTATION BARRIERS**

3. Contingency management (CM) is a behavioral intervention. This intervention principle suggests that behaviours are more likely to increase in frequency if they are motivated and decrease in frequency if they are negatively de-motivated. Implementation involves systematic delivery of rewards or punishment focusing on the occurrence of the target response. CM interventions have substantive evidence of efﬁcacy in positively modifying a variety of patient behaviours. (All interviewees)

4. Tell me what you know about this intervention (Contingency management)

5. Why in your opinion, this intervention (CM) has not been used in Tanzania methadone clinics?

6. How useful could CM be at this clinic?

7. How difficult would it be to implement CM at this clinic?

8. In implementing contingency management, what challenges or barriers would you expect from the MZRH administration?

9. In implementing contingency management, what challenges or barriers would you expect from the methadone service users?

10. In implementing contingency management, what challenges or barriers would you expect from the methadone service providers?

**CONTIGENCY MANAGEMENT-USERS & PROVIDERS PREFERANCES**

11. CM can be conducted using two main incentive categories, money and materials. Which category would you prefer to be used?

12. Please, indicate reasons for your response in question 11?
