# Supplementary material for: Barriers to implementing contingency management at a methadone treatment clinic: A qualitative study at a tertiary hospital in Tanzania
Source: PLoS One. 2025 Mar 3;20(3):e0314168. doi: 10.1371/journal.pone.0314168 (PMC11875383; doi:10.1371/journal.pone.0314168)
Supplement: S3 File — (DOCX) [file pone.0314168.s003.docx]

| Interpersonal factors  Knowledge | Please describe what you understand by contingency management for methadone users  Based on your understanding, do you know any advantages of this intervention? |
| --- | --- |
| Beliefs and functional meanings  Skills | Why do you think this intervention has not been popular in Tanzanian services?  Could you tell how best this intervention can be implemented?  What do you think institutions delivering the methadone services can do to get used to CM? |
| Social economic factors  Costs | What average costs values would you propose for the intervention to be implementable? |
| Policy and managerial issues | What types of policy issues would be appropriate to facilitate implementation of CM? |
| Other related issues | Any relevant questions related to contingency management related to methadone users and services? |

**Focus group Interview guide**

*Assessing barriers on implementation of contingency management for methadone users at Mbeya Zonal Referral Hospital.*
