# Supplementary material for: Barriers to implementing contingency management at a methadone treatment clinic: A qualitative study at a tertiary hospital in Tanzania
Source: PLoS One. 2025 Mar 3;20(3):e0314168. doi: 10.1371/journal.pone.0314168 (PMC11875383; doi:10.1371/journal.pone.0314168)
Supplement: S4 File — (DOCX) [file pone.0314168.s004.docx]

**Supporting information:** **Data sets for Barriers to implementing Contingency management at a methadone treatment clinic. A qualitative study at a tertiary Hospital in Tanzania**

**IN-DEPTH INTERVIEW DATA SETS**

**PARTICIPANT NO. SIX (22 YEARS----- Z0000025-VLC media player) IM5**

**Interviewer:** After reading the consent form to you, are you ready to continue to participate in this study?

**Participant**: Yes, I am ready

**Interviewer: Ok, you are warmly welcome I will continue asking you questions. I would like to know your age**

**Participant**: I am 22 years

**Interviewer: (Twenty-two years) Your marital status?**

**Participant:** Not married

**Interviewer: (Not married), Who are you living with?**

**Participant:** I am staying with parents

**Investigator: (with parents) … What is your religion?**

**Participant:** Christian

**Investigator: (Christian)… What is your highest level of education?**

**Participant:** Certificate level

**Investigator: (Certificate level) …How many years did you spend to study in this level of your education?**

**Participant:** One year

**Investigator: (One year) What is your source of income?**

**Participant:** Business

**Interviewer: … If you are employed name the job that makes you get salary**

**Participant…**I am not employed

**Interviewer:** You’re not employed

**Participant:** Yes

**Interviewer: Well, no problem, for how long have you been providing methadone services here to date?**

**Participant:** This is a third Month

**Interviewer**: **(Third Month), mention the difficulties and challenges that you encounter about methadone services.**

**Participant…….** the big challenge is for these methadone users **(Interviewer: Mmh**) I can say that they do not follow the preset instructions, (**interviewer**: they do not follow the rules, mmh) such as how they put on attire **(Interviewer**: Mmh**) time** to arrive, they are instructed that the end of service is this time, but they come late

**Interviewer:** (reciting: they come late)

**Interviewer: Perfect, now I will read to you, the obstacles of implementing motivation to methadone users. I will read to you so that you can understand a little bit, then I will ask you some questions below which is the major aim of our study. Motivation to methadone users is the intervention of behavior, the procedures of this intervention suggest that, behavior change to be more positive when there is a positive motivation and declines to be more negative if they will be provided with negative motivation. The implementation involves the system of providing congratulation or punishment with a focus on the occurrence of the expected response. Motivation to methadone users contains substantial evidence in the modification of different behaviors of patients. Now I want you to tell me what you understand by motivation to methadone users as part of behavior modification?**

**Participant:** May be…. what I understand is that, if they will be given those…. any motivation, it will make them change to a greater extend.

**Interviewer: Aaah that means, you believe that motivation will bring about changes**

**Participant:** Yes

**Interviewer: Mmh, but also according to your views why this intervention is not being used in methadone clinics in Tanzania. Why do you think we are not using these methods?**

**Participant………**(silence)

**Interviewer: It means, what do you guess to be the reasons for not using this method to motivate methadone patients?**

**Participant:** May be because of themselves, the way they bring difficulties in working

**Interviewer: ( Mmh difficulties in working), Another thing?**

**Participant……**…(silence)

**Interviewer: any other thing that you think as to why Tanzanian clinics do not use this method of giving motivation?**

**Participant**: I think may be because of methadone users themselves do not have a good cooperation with workers

**Interviewer: Mmh cooperation**

**Interviewer: Well, what do you think if the motivation comes, that is now the motivation is here and patients are being given; such as a person who came early at the clinic is given some money? What do you think will be the usefulness to the clinic if we will start giving motivation?**

**Participant:** it will change behaviors to some people

**Interviewer: Mmh, another thing? Another thing which will be helpful at the clinic?**

**Participant:** It will pronounce the good reputation of the relevant clinic

**Interviewer:** **Reputation of clinic, another thing?**

**Participant**: …(silence)

**Interviewer: OK, if we will start providing these motivations what do you think will be the difficulties in implementing this motivation to methadone users in this clinic, what difficulties we will get in providing motivation to patients in this clinic?**

**Participant:** The difficulties will be there if methadone users will not show cooperation

**Interviewer: Mmh not showing cooperation, another thing?**

**Participant:** I think it is only that.

**Interviewer: Mmh**

**Interviewer: Again, in the implementation of motivation to methadone users what are the challenges or obstacles do you expect to come from the hospital leaders, that is, now we are supposed to start providing motivation what do you think will be the challenges from the hospital administration?**

**Participant:** Follow up

**Interviewer: Follow up, another thing?**

**Participant:** Only that

**Interviewer: Only that**

**Interviewer: In the implementation of the provision of motivation to methadone users for the aim of modifying their behavior, what are the challenges or obstacles we expect to get from the user of methadone services, that is now the patients who use methadone, which challenges do we expect to get from them if these motivations will come to be given when they do well?**

**Participant:** …the challenge which I see is that of personal hatred for those who will not be in good understanding.

**Interviewer: ahaa, personal hatred, another thing?**

**Participant…** (silence)

**Interviewer: Another thing that you see is a challenge that will come from patients?**

**Participant**: …Mmm!...(Silence)

**Interviewer: None eeh, Ok**

**Interviewer**: **In the implementation of this intervention what challenges or obstacles do you expect to come from the methadone service providers, that is what challenges may come from them?**

**Participant:** To provide education to the users

**Interviewer: Mmm, to provide education, another thing?**

**Participant…….** (silence)

**Interviewer: Only those**

**Participant:** Yes

**Interviewer: OK well, but also this interventions can be provided by using two types of motivation, you can give patients money or stuffs, let us say those who will come early we will give them sugar or may be those who are not taking other substance to mix with methadone we will giving them money, now in this section, you as a methadone worker what do you suggest money or stuffs?**

**Participant:** stuffs

**Interviewer: Mmh, now, why do you see stuffs to be good for our patients to be given?**

**Participant:** They will bring about development for them rather than giving them money

**Interviewer: Mmh, development**

**Interviewer: Thank you our questions end here, thank you for your cooperation. Thank you very much.**

**Participant:** Thank you

**Interviewer: Thanks**

**End**

**PARTICIPANT NO. EIGHT (28 YEARS------ Z0000033 VLC media player) IM4**

**Interviewer:** You are welcome to participate in this study, I will be asking you questions according to the study questionnaire of this study on the implementation of motivation to methadone users.

**Participant:** Thanks

**Interviewer: Mh, I would like to know your age**

**Participant:** I am 28 years old

**Interviewer:** **your marital status?**

**Participant:** Single

**Interviewer:** **With whom are you living with?**

**Participant:** I am living with a family, my siblings

**Interviewer:** **Your religion affiliations?**

**Participant:** I am Christian

**Interviewer: What is your highest level of education?**

**Participant:** Degree

**Interviewer: How many years did you spend to attain your highest level of education?**

**Participant:** Five years

**Interviewer**: Mh, what is your source of income?

**Participant:** Formal employment

**Interviewer:** **I would like to know, for how long you have been providing methadone services?**

**Participant:** Mmh, it is one year

**Interviewer: It is one year, what challenges do you know for methadone clinic?**

**Participant:** Mmh (laughs) the challenges I know…, I can put them into two groups; the practical challenges like the challenges of the institution and challenges of patients in the institution. The challenge from the institution however it is in the process of solving, challenge of motivation to workers, but the challenges of patients or customers are for them to come late, behavioral changes, not being serious, their bad language, and other things like these.

**Interviewer: OK, fine, no problem. Now I will read to you a few explanations about the implementation of motivation to methadone users then, I will ask you a few questions. We say that motivation to methadone users is the behavioral intervention, the procedure of this intervention suggests that behavior change to be more positive when there is positive motivation and become more less and becomes negative if the motivation is negative to our patients. The implementation includes the provision of congratulation or punishment to the targeted people, for the aim of the occurrence of the expected response. Motivation for methadone users has a great evidence that it has been making various behavioral changes to patients. Now I would like to ask you these questions as a doctor, what do you understand about the provision of motivation as part of behavioral change?**

**Participant:** Mmh, let me say that, not to people who use methadone alone, but to different groups of people, motivation is something that a person is given, it can be in the form of congratulation, in order to make him do much better for the things he was doing before. It can be in the positive way or negative but the aim is to get the results which are good.

**Interviewer: OK, fine, according to your understandings why this method is not practiced in many methadone clinics?**

**Participant:** I think, first... what I can immediately say is eeh… let us say awareness, when there is no awareness, first of all motivation is the issue of administration that is the sponsor. I want to talk more about positive motivation for example, at our clinic here we can identify clients who are doing well, they are no longer using substances of abuse, UDS is clear, maybe there could be a way to give them motivation as gifts or whatever like T shirts, that they are doing well and it will be a memory to them, that I did well in methadone and got this thing, and when others see the one who has done well, they can do well also. But for stakeholders/funding partners, that issue is not in the budget for now. Therefore, they are just issuing to implement. That to the funding partner is the part to be added there, that is all, and it will be applicable.

**Interviewer: Mmh, OK, fine. And another thing, if we decide to provide motivation for people who are affected by the substances of abuse do you think that it will be useful to the clinic?**

**Participant:** Yes

**Interviewer: It will be useful, what kind of usefulness?**

**Participant:** Yeah, first, to add the positive outcomes, it will be bigger because you motivate a person, not in methadone alone even somewhere else, if you give a person a good present because he has done a good thing you will push him do much better and it will not be only for the person who has been given the present, others also will follow there. Therefore, it will help us even to reduce the number of defaulters because if people will hear that there, there is a motivation many people would like to join but also will increase the number of graduate and also the number of clients will add up.

**Interviewer: Well, now in the implementation of the provision of motivation. What do you think will be the difficulties in implementing motivation at the methadone clinic?**

**Participant:** Yeah, the difficulties that will come out is from clients, other clients to think that why are you giving to this one, why this one and not somebody else, and this will happen if clients have no awareness, and the lack of awareness will happen if clients will not have a good understanding, but I think there will not be much challenges apart from that.

**Interviewer: Well, in implementing motivation to methadone users what are the challenges or obstacles you expect to come from Mbeya Zonal Referral Hospital administration?**

**Participant:** I do not think that there would be challenges from the hospital administration unless if we will need the hospital administration to do that work of providing motivation, the challenge which I see can be of finance, that is the issue of money, if you send there the request that we want to start giving motivation of this and that type, so we need the Hospital to contribute this amount of money, I do not know how the administration will perceive it, it can be a challenge because they can say they have other priorities of other vital things compared to what we are asking for, but if we go to the funding partners may be the challenge will be minor.

**Interviewer:** **OK, well, in the implantation of motivation to methadone users for the aim of changing behavior what obstacles or challenges do you expect to come from the methadone services users that means clients?**

**Participant:** I have already answered that question

**Interviewer:** **You have answered it, it is a repetition eeeh!, OK, well, and in the implementation of the administration of this intervention, what challenges or obstacles do you expect to come from the methadone service providers**?

**Participant:** Eeeh, challenges form methadone services providers I do not think that there would be any challenge from the methadone service providers.

**Interviewer:** **Well, this intervention can be implemented by using two types of motivation; money or things, according to your opinion, which one would you suggest to be used**.

**Participant:** Straight, things and not money.

**Interviewer:** **Mh, why things and not money?**

**Participant:** Yeaah, there is….eeh about money eeh, everybody likes money, there is no one who does not like to have money, therefore if you say you will be issuing money to a person who has done this and that, it can bring about bias especially to clients, and it will create conflicts because they will start saying it was like this and that and why this one is given and that one is not given and what and what, but also the environment itself to handle money, the bias can happen even to staff because if your are given money is a sensitive thing but it is different from things, may be this thing and that thing at least

**Interviewer: OK, fine, thank you for the good answers, I think we have reached the end of our questionnaire, thank you for participating.**

**Participant:** Thanks

**End of Interview**

**PARTICIPANT NO ONE (28YEARS--------- Z0000026 VLC media player) IM 1**

**Interviewer**: Thank you for listening while I was reading the consent form, are you ready to participate in in this study?

**Participant**: Yes

**Interviewer:** You are ready, thank you, now I will start asking you questions from the questionnaire.

**Interviewer: I would like to know your age**

**Participant**: I am 28 years old

**Interviewer**: **You are a male, your marital status?**

**Participant**: I am married

**Interviewer**: **Married, who are you living with?**

**Participant**: I am living with my spouse, I have a wife

**Interviewer**: **Do you have children?**

**Participant**: I have one child

**Interviewer**: **OK, You’re Religion?**

**Participant**: Christian

**Interviewer**: **OK, what is your highest level of education?**

**Participant**: Is… a diploma

**Interviewer: A diploma, how many years did you spend to achieve your highest level of education?**

**Participant:** Three years

**Interviewer**: **Mh . I would like to know your source of income**

**Participant:** Mmmh, the source of income is salary, but also the entrepreneurship

**Interviewer**: **Salary, But also, I would like to know, for how long you have been providing methadone services here.**

**Participant:** One year,. One year and like…six months

**Interviewer**: **Mmh, Ok, now you have been in methadone services for one year and six months what do you think are the challenges related to methadone services?**

**Participant:** There are many challenges, aaaah…. there is a challenge of long distance

**Interviewer:** **Distance, distance for service providers or patient?**

**Participant:** Long distance for patients

**Interviewer**: **Mmmh**

**Participant:** To get transport fair, that means daily subsistence costs

**Interviewer:** **Mh, Subsistence costs, another thing?**

**Participant:** But others is the limited understanding (**Interviewer:** **aah, limited understanding**) of the service provided and certainty of quick recovery **(Interviewer: quick recovery)** many of them are used to take medication for two to three days and they recover but this one takes a bit longer.

**Interviewer**: **Mh, OK, Now thank you for your answers, but now I will explain this intervention of providing motivation for methadone users and below I will ask you some questions so that you can help me. We say barriers in the implementation of motivation for methadone users. Motivation for methadone users is an intervention of behavior, practices of this intervention suggest that behavior change to be more positive when there is a positive motivation and it is reduced more and become negative if they receive negative motivation. The implementation includes the system of offering congratulations or punishment aiming at the occurrence of the expected response. Motivation for methadone users has a great evidence of effectiveness in changing various behaviours of patients. Now I would like you tell me what do you understand about motivation for methadone users as part of changing behavior.**

**Participant:** Aah…, motivation for anybody apart from patients is something that help us a lot to be able to make our goals, because motivation is like a goal that is, today there is something to be given after something has been done, so it adds diligence **(Investigator: diligence)** it increases the morale in reaching the target because of that preset motivation, **(Interviewer**: OK) eeh, but also it may not be mentioned and it can be like a surprise eeh… then it can be done, and a person can do something knowing that “I am doing this, and in any day It can happen also for me to get something” so it provides a continuation for the person to do something in order to protect his/her dignity **(Interviewer: Mmh OK…)**

**Interviewer:** **According to your views why do you think this intervention is not being used in methadone clinics in Tanzania?**

**Participant:** Aaaah…., I think the big challenge is budget **(investigator: Budget),** budget is a challenge because we like to do that, but when do we do that, and we will do it to how many clients, it will be a challenge, you can do that and it can happen only once, but there is no specific budget may be if a person can do that by himself/herself in the ways he/she likes.

**Interviewer:** **Another barrier?**

**Participant:** Aaaah another barrier I think is planning, that is, if as a clinic we can plan that for this year we will give a certain amount of motivation eeh, it is easy for the person to say that three people who have come earlier today I will do something and it does not hurt eeh, we do not need to a have a very huge fund, eeh.., therefore planning, and even ourselves we are not organized to have the routine of this kind, but it is a good routine that can provide us with client sustenance in receiving services.

**Interviewer**: **Well, and what is the benefit in this clinic, what do you think will be the benefits this clinic will achieve in providing motivation to clients?**

**Participant:** Aaah the aim of having clinic is for people to have positive behaviours **(investigator: Positive)** yeah, and if this is the major goal that means that, if you can make something that will make them to…to …walk in the positive line to reach their goal, they fulfill your goal and their goal as well, eeh, therefore both of you will be in a win win situation, yeah, therefore it is something which is a good.

**Interviewe**r: **Yeah, so you mean that the goals will be met**

**Participant**: Goals will be met

**Interviewer: Mh, Well, thank you for the answers, but also, I would like to know, what difficulties do you think will occur if we will start providing motivation to patients, in implementing motivation to methadone users in this clinic?**

**Participant:** The difficulties which can emerge is the selection of who is to be given that motivation.

**Interviewer:** **who is to be given the motivation?**

**Participant**: but also, the estimates of the motivation, that you may became astonished that you have given the motivation in January and another one in December….!, and in between they can stay for a very long time without being given motivation, so there will be a delay also.

**Interviewer Well, in the implementation of motivation for methadone users what kind of challenges or kind of barriers do you expect from MZRH hospital administration?**

**Participant:** Aaaah… the challenges which may arise from the leaders may be when you ask for budget or fund, and you find that there are challenges and other priority which are bigger than this one, because this one is a motivation that you are doing, but in the institution, there are many things to do with squeezed budget so you can get that barrier despite that it is a good plan but you may not be able to do that.

**Interviewer**: **OK, Well, but also in implementing motivation to methadone users for the aim of changing behaviors which challenges or obstacles do you expect to get from the users of methadone services?**

**Participant:** The users…eeh what I see is for themselves how they receive it, there are those who can receive it well but there are others who can see it like ah, they are giving us because of something eeh, therefore, the aim of the service which they receive might be weakened because they use methadone so that they can be recovered and hence they can take the motivation negatively.

**Interviewer:** **this can make them to stay for a very long time**

**Participant:** Yes

**Interviewer**: **Well, in fulfilling the role of providing this motivation what challenges or obstacles do you expect from the methadone service providers?**

**Participant:** Eeeeh… service providers can face obstacles as I said before that, you ask for the fund and you do not get it on time and now who will volunteer? (laughs) who is to do that?

**Interviewer: OK.**

**Participant:** Eeh or in fulfilling that, in fulfilling the program of giving motivation will it be coming correctly? Or equally without favoritism? Yeah, therefore this is a challenge that might happen.

**Interviewer:** **Ok no problem…. but now……thank you for answering my questions, but I would like to say that this motivation can be done by using two forms of motivation; there is money motivation and item motivation, I would like to know from you as a provider of methadone clinic here, what would you prefer to be used between money or items?**

**Participant:** Aaaaah, there are circumstances they can be used, but mainly for people of that kind, the clients we have, things can be better than money because of the type…. nature of life they lived and their spending eeeh, so money to them may not be of value because they used to spend a lot of money for substances abuse therefore even the discipline of money will not be there, but if you give him/her something he will value it and can also take care of it or can use it as a capital to do something rather than giving him/her cash.

**Interviewer**: **Well, thank you for participating and answering questions well, thank you, we have reached the end.**

**Participant:** Thank you

**End of Interview**

**PARTICIPANT NO THREE (30 YEARS ----------Z0000022 VLC media player) IM 3**

**Interviewer**: I would like to know if you are ready to participate in this study after reading to you the consent form.

**Participant**: Yes, let us proceed

**Interviewer:** Thank you for accepting to continue with this study and for accepting to cooperate, I will be asking you some questions and we have several questions and I am requesting you to respond in the way I am asking you.

**Interviewer: I would like to know how old you are**

**Participant**: I am 30 years old

**Interviewer: I would like to know your marital status**

**Participant**: I am married

**Interviewer: Who do you live with at home?**

**Participant:** My wife

**Interviewer: What is your religion?**

**Participant: C**hristian

**Interviewer: What is your highest level of education?**

**Participant:** I completed form four

**Interviewer: Mmh form four. How many years did you spend to attain this level of education?**

**Participant:** I spent five years; it means I repeated one year

**Interviewer: what is the source of income for you?**

**Participant:** Personally I… maybe I can mention those related but I am a business man

**Interviewer: Ok, and…. for how long have you been using methadone services until now?**

**Participant:** Up to this moment this is a second year

**Interviewer:** **mention the difficulties and challenges related to methadone services.**

**Participant:** Like how many?

**Interviewer: Any, two, three which you know that are challenges in methadone services**

**Participant:** The first one which I see, many patients who are coming for methadone services, issues of transport fare to reach at the methadone center**.**

**Interviewer: Mmh, any other challenge?**

**Participant:** Another challenge is the relationship between……. especially to pharmacists who are dispensing and patients, the relationship is not good at all**.**

**Interviewer: The relationship with service providers, another thing?**

**Participant:** Number three… this one is with addicts themselves, the way to do what….to understand the importance of treatment many of them do not follow the treatment as is supposed to be, they are forging as if a person does not understand the importance of receiving methadone.

**Interviewer: Mmh, another thing?**

**Participant:** Another difficulties that we get is the law issues

**Interviewer: Aah law**

**Participant:** Eeeh!

**Interviewer: The legal cases you get?**

**Participant:** The legal cases that we encounter most often.

**Interviewer: Thank you for your answers but also, I will explain to you the obstacles in the implementation of motivation to methadone users by giving you a short explanation from this study we are conducting. Motivation to methadone users is the intervention of treatment. The procedures for this intervention suggest that, behavior changes to be positive when there is motivation and becomes less positive when they receive negative motivation. The implementation includes the offering of congratulations or punishment aiming at expected positive response outcome. Motivation to methadone users signifies and it has a big evidence that it is effective in changing various behavior of patients, here we are talking about giving punishments or congratulations, do you understand me?** (Participant: Yes) **for example if a person is tested and found to be free from heroin can be given a bus fare, or a person has done something wrong then can be punished, (Participant reciting: punishment) so we expect that these things, like those who come early in the morning they are given bus fare so we expect these things to bring about positive outcome in the behaviors of those who receive services** (Participant: the general running of clinic...) **Now with this explanation I have given you I will ask you a few questions.**

**Interviewer: Tell me what do you understand about motivation to methadone users as part of changing behavior?**

**Participant**: For me the way I see… for these users, motivation as motivation when it will start to be provided, and for behaviors of users that I had when I started to smoke until now, a customer is a changeable person according to the environment (**investigator reciting: Environment**) Eee! so, if there will motivation as the previous examples you have given me, I guess we can make positive behaviors (Interviewer**: Mm**) Even if a person can be acting but later, we are sure one thing can change because you cannot deceive to come to take methadone at 07:00 am then tomorrow You come at 10:00 (laughs) while you know that there is a certain motivation. So, I strongly believe that it will build a positive behavior.

**Interviewer: (Reciting: It will build a positive attitude)** **Well, and according to your views why do you think this… this issue of providing motivation in methadone clinics in Tanzania, is it being applied or not?**

**Participant:** Here at Mbeya for our program since it started it is not applied

**interviewer: (Reciting: not applied) and that of giving punishment?**

**Participant:** Punishment is applied

**Interviewer**: **It is applied, but is it applied exceedingly or slightly?**

**Participant:** It is only average they are not much to it.

**Interviewer: Do you think if we will use this method of providing motivation to methadone patients what will be the benefits for this clinic?**

**Participant:** I think we will increase the number of our patients

**Interviewer: (Recitation: Number of patients) Another thing?**

**Participant:** Another thing we will change the negative behaviors because there are people who steal so that they could get bus fair, or get this eeh, so we will strengthen patients and their attendance will also improve. Attendance will be good.

**Interviewer: Mm, Another thing?**

**Participant**: The relationship also will be good between aaa…. we will improve relationship

**Interviewer: (reciting: relationship) Another?**

**Participant:** I have only that.

**Interviewer: That is what you had, thank you for you good arrangement on the way you have answered questions, also what will be the difficulties in implementing motivation to methadone users in this clinic, it means what difficulties in this clinic in implementing the motivation, what difficulties are likely to occur in implementing this**?

**Participant**: The difficulties which might happen in implementing this, the difficult may happen when….. let me think………. I think the difficulty in this, we can get from the addicts themselves that is, people might start giving wrong reasons, that is if we give transport fair for those who stay away a person may deceive where is his/her place of residence you see?

**Interviewer: so that he can get the motivation**

**Participant:** Eee so that he can get the motivation

**Interviewer: Mmh, Another thing?**

**Participant:** Another thing, I also think for us as I know the way we are there will be complaints among ourselves that there are others who are favored, like a certain favoritism for those who receive motivation

**Interviewer: Mmmh favouratism, another thing**

**Participant**: Another thing what I see there... For negative change I think these two things will be very disturbing.

**Interviewer**: **In the implementation of motivation to methadone users what challenges or obstacles do you expect from our hospital leadership of Mbeya Zonal Referral Hospital, challenges at the hospital side?**

**Participant:** The challenges that the hospital can get?

**Interviewer: Mmh Challenges that the clinic will get from the hospital**

**Participant:** Mmh, Mmh not to receive the money on time

**Interviewer: Aah, the money not to reach on time, another?**

**Interviewer**: **Another, can you mention another thing?**

**Participant**: Another thing what I see is the honesty among workers themselves.

**Interviewer: (repeating: Honesty among workers)**

**Participant:** Eeeeeh….

**Interviewer: Another thing?**

**Participant:** Aaaaah…I see only these my mother.

**Interviewer: Well, In the implementation of motivation to methadone user for the aim of changing behavior what challenges or obstacles do you expect from the users of methadone services?**

**Participant**: Repeat please

**Interviewer: Well, In the implementation of motivation to methadone users for the aim of changing behavior what challenges or obstacles do you expect from the users of methadone services?**

**Participant:** Many of them will be pretending to wear the character, the changed character while they have not changed

**Investigator: … Mmh, wearing an unrealistic character**

**Participant**: Eeeh

**Interviewer**: **And in the supervision of the implementation of this intervention what are the challenges or obstacles do you expect to get from methadone service providers, which means service providers of methadone, here, what kind of challenges that will come from them that can be seen?**

**Participant: ….** Challenges from health providers

**Interviewer**: **Here we are talking about; let us say the issue of motivation has already come, and when it has come it will involve people who use methadone and service providers, now we say what challenges as you see, that these challenges will come from the workers.**

**Participant**: I think this question I can compare with the certain answers I gave here before that we go back to the issue of honesty among themselves you see?

**Interviewer: Mmh, honesty Mmh, another thing?**

**Participant:** I said honesty and what?

**Interviewer: Here I think you said honesty and not to receive the money on time for the questions you answered before.**

**Participant:** Eeeheee, but also here you may wonder to see that the money reached on time to the staff but to reach to the patients it is a challenge now by itself

**Interviewer: Mmh, OK Eeeh. Another thing?**

**Participant:** Ahaa, I see only these

**Interviewer: Well, now we go to another question which says this intervention can be implemented by using two types of motivation whereby there is money motivation and items motivation, now you as a patient if they ask you that we want to give motivation here do you need money or items, what is your suggestion?**

**Participant:** For me personally I prefer money

**Interviewer**: **You will prefer money, and you think what reasons make you see that money will be better in providing motivation?**

**Participant:** I see money is better in providing motivation because it is also easier to preserve.

**Interviewer: To preserve mm, another thing?**

**Participant:** Eheee…the easily availability of money on time, because nowadays we have

money services, eeeh.

**Interviewer: Mmh, another thing that makes you see money is the best idea?**

**Participant**: It is easier to confirm between me who is given the money that, this one is being given let us say 10,000 and this one has deducted 10,000 which is different from things, Eeeh**.**

**Interviewer: Things that you do not know the cost**

**Participant**: Eeeeh, I will not know what, and what came out there I will be brought things like take this soap and rice may be…, Eeeeh**.**

**Interviewer:** Well, another thing?

**Participant:** No

**Interviewer**: **We have reached the end of our questions, thank you very much for you cooperation. Thank you very much.**

**Participant:** No problem, thank you too for involving me in this program

**The End**

**32 years PARTICIPANT NO. SEVEN (32 YEARS-------Z0000032 VLC media player) IF4**

**Interviewer:** For what I have just explained to you, are you ready to participate in this study?

**Participant**: I am ready, no problem.

**Interviewer:** OK, thank you for your willingness.

**Interviewer: I would like to know your age**

**Participant:** I am 32 years old

**Interviewer: Your gender?**

**Participant:** Female

**Interviewer: Your marital status?**

**Participant: I** am single

**Interviewer**: W**hom are you living with?**

**Participant**: I am living alone

**Interviewer: Your religion affiliations?**

**Participant**: Christian

**Interviewer: What is your highest level of education?**

**Participant**: I have a degree in sociology

**Interviewer: How many years did you spend to study your highest level of education?**

**Participant**: Three years

**Interviewer: What is you source of income?**

**Participant:** Employment but also, I have another business somewhere there.

**Interviewer: You have another business, Ok, thank you very much. For how long have you been providing methadone services until now?**

**Participant:** Mmmh, since 2017, it is three years**.**

**Interviewer: Mention the difficulties or challenges related to methadone services**

**Participant:** Challenges which I encounter, first is to deal with these clients, is difficulties due to their nature and the environment in which they come from, and again we did not know them, we did not stay with them to know their behaviors, we have met them here, so it becomes a challenge, you are faced with an abusive language, bad language, you are faced with obstacles and you can be beaten sometimes if you are not careful, therefore all those are challenges**.**

**Interviewer: Ok, well, thank you for your explanations, now I will read to you the obstacles of motivation for methadone users, then later on, I will ask you some questions. Motivation to methadone users is the intervention of behavior, the discipline of this intervention suggests that behaviour change to be positive when there is a positive motivation and diminishes and becomes negative when you are provided with negative motivation. The implementation includes the system of providing congratulations or punishment aiming at the occurrence of the expected response. Motivation to methadone users has great evidence of effectiveness in changing various behaviours of patients, now, tell me what you understand about motivation to methadone users as part of changing behavior.**

**Participant:** Sorry, repeat your question

**Interviewer: That means, I would like to know, what you understand about motivation to methadone users a part of changing their behaviour.**

**Participant:** OK, the way I understand motivation to methadone users, for example we can observe those clients with good progress since he/she started to attend clinic or taking methadone up to this time, and we find that his/her progress is good, therefore you can check, the motivation that you can give, mmh, you can look for any gift for him/her then you can give it to encourage him to continue well with clinic, his attendance you can give him a gift it can be money, aaah…another motivation you can offer them outing, may be they are five or six you can offer them outing and you tell them that you people have impressed me because of one, two three…keep it up.

**Interviewer**: **Mh, well, now according to your views why these interventions are not practiced in many methadone clinics in Tanzania?**

**Participant**: Aaah the first thing I can say is understanding, or there is no awakening when we start with us methadone staff who are working or providing services to these people, that means we do not take this issue seriously mmh, it does not mean that we do not see it, we see their development but we have some sort of heaviness on this issue**.**

**Interviewer**: **Mh**

**Participant:** But another reason I can say... now I am not sure with it but there is no…. there is no…… that means there is no money to fulfill the needs of these people such as motivation, for example those who are graduating in which way are you going to congratulate them, maybe there is no that budget or no money.

**Interviewer**: **Mmh**

**Participant**: But another thing I can just say… aah…, mmh…, it is a matter of not making decision, it does not mean that there is no money, it is just that they do not like to make decision, they understand, they know, but they do not like to do such a thing.

**Interviewer: Yes, decision. OK, well, now do you think if it happens may be this method of providing motivation starts to be implemented in methadone clinics what will be the benefits in methadone clinics?**

**Participant:** First of all, it will change the behaviours of these clients we are serving, it will greatly change, there would be a significant change starting from …let us say the attendance, behaviour change; in behavioral change there are many things, many things will change because in his mind he has the information that there is this and that thing for getting motivated, so I am also struggling to get there.

**Interviewer: Mh, well and… what will be the difficulties in the implementation of providing motivation in this clinic?**

**Participant:** Repeat again the question

**Interviewer: It means the difficulties for example, it has happened that today we have started to provide motivation or you have started to provide motivation in this clinic, what do you think will be the difficulties which will happen in this clinic when motivation starts to be provided for implementation?**

**Participant:** Mmh, the difficulty which I can say quickly is the complains from the clients that you will be seen as you favor others and you do not do the same to others you do not give those motivation to others, therefore be ready to receive the complains.

**Interviewer: Complains, Mh, OK, well.**

**Interviewer:…….and in the implementation of motivation to methadone users what challenges or obstacles do you expect to come from MZRH administration?**

**Participant**: Mmmh, may be time, the time to start implementing, it can take long time, I become frightened because they must have that information, therefore up to the time you are allowed to start implementing that issue it may take long time.

**Interviewer**: **Long time, well, another thing?**

**Participant:** Another thing…may be let us say the money have been sent for that motivation if it will pass through hospital, we have to expect…, but I am not sure they can be reduced for the reasons I do not know; you may expect 10 million and you will be given 6 million.

**Interviewer: That means the budget will not be real**

**Participant:** Enhee

**Interviewer: OK fine.**

**Interviewer: In the implementation of motivation to methadone users for the aim of changing behaviours what challenges or obstacles do you expect from the users of methadone services that means the clients, what challenges do you expect from them.**

**Participant:** Repeat again

**Interviewer: That means in the implementation of motivation what challenges will arise from the clients, that clients will generate those challenges while we continue with the provision of motivation**.

**Participant**: Mh, one as I said that complains

**Interviewer: Complains**

**Participant**: We have to expect to receive complains, but …second you will also receive abusive language,

**Interviewer**: **Abusive language**

**Participant:** But third be ready also for disturbance that means either to the social officer office or psychologist you might find yourself everyday receiving people who are asking that they have problems begging you to put them in this way or that way such kind of disturbances.

**Interviewer**: **Mh, OK, fine, and in the implementation of administering this intervention which challenges or obstacles do you expect from methadone service providers?**

**Participant**: In supervising

**Interviewer**: **Yes, in supervising**

**Participant**: Mmh, the challenge that may happen, which I see that will be big is if everyone would like to be the supervisor of providing the motivation**.**

**Interviewer**: **In providing motivation, Mmh, well.**

**Interviewer: …and in this intervention it is possible to be implemented by using two types of motivation money or things, I am asking you to suggest one thing, which one do you think will be useful between money or things?**

**Participant:** Mmh, What I will suggest is money, because……

**Interviewer: Mmh, why do you think money?**

**Participant**: Because if you say you give a person something, maybe he does not need it at that time, but if you give hiM money may be he was thinking of opening a certain business even if the money is in small amount, he will start with it a small capital to start his/her business.

**Interviewer: OK, well, thank you, thank you for your cooperation from the beginning to the end, I think here we have reached at the end of our questionnaire. Thank you very much.**

**Participant**: Thank you too.

**PARTICCIPANT NO. FOUR (34 YEARS PT--------Z0000023 VLC media player) IF1**

**Interviewer:** I think you have listened to what I have explained to you to ask your consent to participate. Now I am listening to you, are you ready to participate?

**Participant:** Yes, I am ready

**Interviewer:** Ok, thank you very much, now you will sign here and then we will continue with the interrogation

**Participant:** Well

**Interviewer: Ok, now I will ask you direct questions to get your information. First, I would like to know how old are you?**

**Participant**: I am 34 years old

**Interviewer: 34 years**

**Interviewer: Your gender is female isn’t?**

**Participant:** Yes

**Interviewer: Mmh, your marital status?**

**Participant:** I am married

**Interviewer: who do you live with at home?**

**Participant**: I am living with children, the whole family is at home, children, husband

**Interviewer: What is your religion?**

**Participant:** Christian

**Interviewer: Mh, what is your highest level of education?**

**Participant:** Form four

**Interviewer: Form four… and how many years did you spend in obtaining your higher education?**

**Participant:** From Form one, I used…. 11 years

**Interviewer: What is your source of income?**

**Participant:** I have a shop business

**Interviewer: Business…if your employed name the job that makes you to have income?**

**Participant**: I am also employed at KIHUMBE organization: (**Interviewer: Mmh**) as a peer educator

**Interviewer: OK, … Well………I would like to know for how long you have been using methadone services to date?**

**Participant:** Three years

**Interviewer:** **Three**

**Participant:** Yes

**Interviewer: Mention any difficulties or challenges related to methadone service**

**Participant:** there are minor hurts resulting from methadone

**Interviewer: Mmh**

**Participant:** Those like…. are those which I feel from the body or out of….?

**Interviewer**: **generally, that is difficulties or challenges which result from methadone services as you know here, maybe we have provided services as we said that here, we provide psychological services, biological services and social services, therefore any challenge.**

**Participant:** All services are good may be the challenge is time

**Interviewer: Time, mmh**

**Participant:** Yeah

**Interviewer: Time, how?**

**Participant:** Time…time which maybe you may come late

**Interviewer:** Mmh

**Participant: I**t is that

**Interviewer: Well, I will read to you the obstacles of implementation of motivation for methadone users, you will listen and then I will ask you a few questions. We say motivation to people who use methadone is an intervention of behavior, the procedures of this intervention suggests that, behavior change to be more positive when there is a positive motivation and behaviors diminishes to become negative if they are given negative motivation. The implementation includes the system of provision of congratulations or punishment with a focus on the occurrence of the expected response, motivation to methadone users contains significant evidence in the modification of different behaviors of patients, now with these explanations, I ask you to answer these questions.**

**Interviewer: Tell me what do you understand about motivation to people who use methadone as part behavior modification?**

**Participant:** Mmh, I see motivation for methadone users can help them to change behavior (**Investigator**: **Mmh**) yeah or to make people to… to have a quick recovery.

**Interviewer: quick recovery, another thing?**

**Participant:** Mmh …. another thing is to stop the use of other types of substance of abuse while in methadone treatment**.**

**Interviewer: Another thing?**

**Participant:** Another thing is….to change the appearance, **(Interviewer: Mmh appearance) ….**to change the attending time.

**Interviewer: Mmh time, another thing?**

**Participant:** Another thing is to change, let us say life in general

**Interviewer: Life in general**

**Interviewer and participant together**: Eeeh

**Interviewer: Ok, in your views why do you think this method is not being used in methadone clinics in Tanzania, the issue of providing motivation, why do you think it is not used in methadone services in Tanzania?**

**Participant**: First of all is…. our government has failed to such a thing because of mmh… I do not know how to put it…. first of all, it will also make the users of methadone …., others may enter in methadone treatment to follow something especially those who are not in treatment because they know there is motivation, knowing that may be when I go there, I will be given money…

**Interviewer: Mmh, Ok I will repeat the question, we say according to your views why do you think this motivation is not used in methadone clinic, what are the reasons that make this thing not to be used?**

**Participant:** (Silence)

**Interviewer: Mmh, as I have repeated that why this thing is not being used in methadone clinics in Tanzania?**

**Participant:** As I have said that may be because our government has no money

**Interviewer: Lack of money**

**Participant:** Lack of money to satisfy this

**Interviewer**: **Mmh, another thing?**

**Participant:** Another thing, I think is also because of lack of support mechanism

**Interviewer: Mmh, or let us say we do not have that culture**

**Participant:** That culture, Yeah

**Interviewer: Mmh, another thing?**

**Participant:** Another thing may be the way the methadone constitution says

**Interviewer: Mmh, constitution**

**Participant:** The constitution has no……...

**Interviewer: Mmh,it has no motivation issues.**

**Interviewer: Ok, well, I am asking you another question, what will be the benefits in this clinic if we will decide to provide motivation at our clinic here, what do you think we will get?**

**Participant:** The benefits are many

**Interviewer: Mmh**

**Participant:** as I said before it will help people to change

**Interviewer: to change**

**Participant:** Yeah, to change to stop the behaviours of using other substances

**Interviewer**: **other substances,**

**Participant:** Yeah, because……

**Interviewer: Because?**

**Participant:** because, let us say, for example may be a person is given motivation because whenever he is tested is found to be free from substances so another person will see that and decide not to use so that he/she can also be motivated**.**

**Interviewer: so that he/she can get motivation, I understand you**

**Participant:** Yeah

**Interviewer: Another thing?**

**Participant:** Another thing is coming early at the clinic

**Interviewer: coming on time, attendance will improve.**

**Interviewer: Mh, another thing?**

**Participant:** Another……. there are many indeed

**Interviewer: Mmh, they are many, in general the service will make a difference**

**Participant:** it will bring about positive changes

**Interviewer: Well, thank you for the question, but what will be the difficulties in the implementation of this motivation to methadone users in this clinic, that means; what do you think will be the difficulties if it is brought here and it is supposed to start, what difficulties will happen in introducing it?**

**Participant:** In initiating it must bring difficulties because everyone will know that he/she has a right to get motivation

**Interviewer**: **Has the right to get the motivation, Mmh, another thing?**

**Participant:** Another thing……I think education… may be education is to be given first and then people be given the motivation

**Interviewer: That means people are to be given education first because the challenge here will be peoples’ understanding**

**Participant:** Understanding

**Interviewer: Ehee, another thing?**

**Participant:** Mmh….

**Interviewer: Ok, well, in implementing motivation to methadone users what challenges or obstacles do you expect from the hospital administration, you know this clinic has workers but it is under the hospital what do you think will be the obstacles in introducing it that might be caused by the hospital administration?**

**Participant:** Obstacles… **(Interviewer**: **Mh) …** Mmmh, obstacles that will be caused I think is…. for instance, the motivation is of money, I know it must be under the hospital so the process will be long to get money on time

**Interviewer: Mmh, another thing?**

**Participant:** Another thing is……(silence)

**Interviewer**: **OK Well, in the implementation of motivation to people who use methadone for the aim of changing behavior what are the challenges or obstacles do you expect to get from the methadone service users, here we were talking about hospital administration but here now we are talking about the users, the targeted of methadone, that what challenges do you expect from them if this motivation services will be provided?**

**Participant:** The great challenge is as I have said is lack of education, therefore the targeted people will be feeling as if they are not given their right **(Interviewer:** **Mh**) those who will not be successful in getting motivation**.**

**Interviewer: Mmh, another thing?**

**Participant:** Another is like…… I think the great challenge is that one.

**Interviewer: It is that one OK.**

**Interviewer**: **In the implementation of management of this intervention what challenges or obstacles do you expect from the methadone service providers, those who give services?**

**Participant:** Mmh, challenges at your side, I do not understand

**Interviewer: The side of methadone services providers, what do you think are the challenges expected to occur in introducing it?.**

**Participant:** The big challenges for service providers, as I said is the issue of time...

**Interviewer:** **Time to get that motivation Mmh, another thing?**

**Participant:** Yeah, time to get motivation.

**Participant:** Second to start…... to start a special procedure for performing tests on people

**Interviewer: Mh, the whole process will take time**

**Participant:** Eeeh

**Interviewer: Another thing?**

**Participant**: Another this is……aah no**.**

**Interviewer and Participant:** Laughs

**Interviewer: OK, this intervention can be implemented by using two types of motivation, money or items, according to your views which one would you suggest to be used, between money or items?**

**Participant:** For me I see money to be much better

**Interviewer: Mh, why do you think money is much better?**

**Participant**: It is much better because others do come for methadone but they do not have money for transport, so the money can help him/her to use as bus fare or other problems like food.

**Interviewer:** **Mh**

**Participant:** Yeah,

**Investigator: Ok, so it will help him/her for food, and bus fare.**

**Participant:** Yeah,

**Investigator: Well. Thank you for participating and answering my questions, thank you very much, have a good day**

**Participant:** Thank you so much

**End**

**PARTICIPANT NO. TWO (34 YEARS-------Z0000027 VLC media player) IM 2**

I**nterviewe**r: Thank you for listening are you ready to continue

**Participant**: Yes

**Interviewer:** Thank you

**Interviewer: Now I will start asking you questions from the questionnaire, first I would like to know your age**

**Participant:** 34 years

**Interviewer**: **Mmh, your marital status?**

**Participant**: I am married

**Interviewer**: **With whom are you staying with at home?**

**Participant:** wife, children.

**Interviewer:** **Your religion?**

**Participant:** Christian

**Interviewer**: **what is your highest level of education?**

**Participant**: Masters

**Interviewer:** **How long did you take to attain your highest level of education?**

**Participant:** What do you mean?

**Interviewerr**: **The years you spend to attain the skills of that level of education**

**Participant:** Masters two years, degree three years

**Interviewer**: **(Mmh, for five years) what is the source of income for you?**

**Participant:** salary

**Interviewer…For how long have you been a leader at the Mbeya Zonal Referral Hospital?**

**Participant**: eight years now

**Interviewer:** **(reciting: Eight years now),** **if it is eight years I hope when the methadone clinic started you were here, we would like to know you as a hospital leader are there any challenges you know from the methadone services**?

**Participant:** Challenges…one perhaps, methadone treatment is donors oriented, So I expect the day donors will go the services will weaken.

**Interviewer**: Mmh, weakening of services

**Interviewer:** **Well, now I will read to you the descriptions on the obstacles of motivation to methadone users and after that I will ask you some questions.**

**Motivation to methadone users is an intervention of behavior, the system of this intervention suggests that behavour changes to be more positive when there is a positive motivation and it is more lessened and becomes negative when they are provided with negative motivation. The implementation includes the system of providing congratulations or punishment aiming at the occurrence of the expected response. Motivation to methadone users has a great evidence of effectiveness in various behaviours of patients. Now with those explanations, I would like you tell me whatever you know about motivation in methadone services, according to your understanding.**

**Participant**: Maybe because in our zone, it is a new thing, ok; therefore, when you try to look at it, the way they provide services you cannot do the comparison with other places whether they give motivation or not, it becomes a bit difficult.

**Interviewer:** **(Reciting: mmh difficult) Well, according to your views why this motivation is not being used in many methadone clinics in Tanzania?**

**Participant:** The first thing is like what I said, when you look at our systems of budgets, we prepare there is no package for methadone services, therefore it is also difficult to implement something which has no budget allocated for it.

**Interviewer:** **…: (Reciting: no budget allocated) Well, what will be the benefits in this clinic in case it will start to be provided, you as a leader, will it be beneficial?**

**Participant**: In the community it will be beneficial because we expect those who use substances to stop, because there are those who use substances, they stop and they start using again, **(interviewer reciting: they start using again)** Eeeh, therefore if a person cannot use for a long time because he knows that if I will do this, I will be motivated so at the end of the day the craving for a substance will go away for good.

**Interviewer: Mmh it will go, well; Thank you, but also, I would like to know in implementing this intervention, what will be the difficulties in employing the provision of motivation for methadone service users in this clinic, that is to our patients, if we want to start giving motivation what difficulties are we going to encounter from the users?**

**Participant:** The difficult of……., first everything which is being introduced should not be in the form of money **(Interviewer reciting: Mmh it should not be in the form of money mmh**) ……...so that services are sustainable, because if you will start it in terms of money there is a time you will have no money that means people will stop doing what they are doing.

**Interviewer: (they may stop, Mmh, yes) Also I would like to know when this service starts to be implemented what are the challenges, we will get from the hospital administration?**

**Participant**: Mmmmh…. May be because it is a new thing, the challenge which may come is the limited budget at our side, it will be a problem because you cannot start incentives without……. because even if you will say it is none financial incentive but you will have to use money **(Interviewer: to get it)** Yeah.

**Interviewer:** **Well, and in the management of implementing this intervention what are challenges or obstacles do you expect from the service providers of methadone clinic, because you are a leader and there are those who provide services, what challenges do we expect to get from them?**

**Participant:** Mmmmh, May be let me talk about services to customers, because in order for you to be able to have many people and to sustain them, you must have good services, therefore if you lack that kind of services, you bring about challenges to those who receive that service……….so it will depend on how you have equipped or trained them**, (interviewer: Ok, well)** because those people who will be coming they come with different understanding, if they come while they are mentally fit and you can explain it is well, but you may be surprised another person will come with his different things…

**Interviewer:** **(they are different) Well, also we directly say that in providing this intervention we have two types of motivation, there is motivation in terms of money and items. I would like to know which one do you prefer however you said earlier that it should be things**

**Participant**: Yes, more incentives

**Interviewer**: **(incentives) Why do you think it should be incentives?**

**Participant:** It is easier to sustain **(Interviewer repeating: easier to sustain)** for example when we say incentives or something which is not money, let us say for example a person who is coming earlier we can do something like a reward to him/her, or because he/she comes early we can say he/she will not be staying in the que, in order to make the person feel valued.

**Interviewer:** Let me thank you my leader for your participation, thank you very much

**Participant:** Thank you

**End**

**PARTICIPANT NO. FIVE (38 YEARS------Z0000024 VLC media player) IF2**

**Interviewer: I would like to know if you have agreed to participate**

**Participant: I have agreed to participate**

**Interviewer:** Thank you very much for agreeing to participate in this study, I am now going to a special questionnaire

**Interviewer: I would like to know, how old are you?**

**Participant:** 38 years

**Interviewer:** **and you are a female**

**Interviewer: Your marital status?**

**Participant:** I am married

**Interviewer: Married, whom are you living with at home?**

**Participant:** Husband and two children

**Interviewer: Well, what is your religion?**

**Participant:** Christian

**Interviewer: A Christian**

**Interviewer: what is your highest level of education?**

**Participant:** Bachelor

**Interviewer: Bachelor…**

**Interviewer: How many years did you spend to achieve your highest level of education?
Participant:** sixteen…... Years

**Interviewer: 16**

**Interviewer: What is your source of income?**

**Participant:** We depend only on salary

**Interviewer: Mh, well. If you are employed name the job that gives you salary**

**Participant: …** I get paid for my skills

**Interviewer: Mh, well, I would also like to know for how long you have been working here at Methadone clinic.**

**Participant:** Two years

**Interviewer**: **Two years**

**Interviewer: Mention the difficulties and challenges related to methadone services that you know… in providing services in methadone.**

**Participant:** The difficulties………... the difficulties we face, our clients are in hurry, they do not like to be seen by psychologists, doctors…, they believe in medication alone**.**

**Interviewer: Mh, Well, now I will read to you the impediments in the implementation of motivation to methadone users, and then below I will ask you questions. We say that motivation to methadone users is an intervention of behavior, the order of this intervention suggests that behavior change to be more positive when there is motivation, and become less and more negative when they receive negative motivation, the implementation involve the system of providing congratulations or punishment with a focus on the occurrence of the expected response. Motivation to people who use methadone has a great evidence has great Evidence for effectiveness**

**in changing various behaviours of patients. Now I will ask you questions here below, according to what I have explained to you. Tell me what do you understand about motivation to methadone users as part of changing behavior. What do you know as motivation according to your opinions?**

**Participant:** What I see…. because our methadone users many of them do not have a specific source of income **(Interviewer repeating:** **income)** so, the provision of motivation will help them may be to get transport fare, **(Interviewer repeating: transport fare mmh)** because many of them are complaining to come here on foot, this is the first one.

**Interviewer**: **Mmh well, according to your understandings why this method is not practiced in methadone clinics in Tanzania? The reasons that make people not to be given motivation.**

**Participant:** The reason…. the first thing maybe it was not in the budget.

**Interviewer: It is not in the budget mh,**

**Participant**: The second thing may be ...mmmh…they fear may be…there will be inappropriate use of motivation **(Interviewer repeating:** **Inappropriate us of motivation mmh,)** that we may not know the exact number of people who are supposed to get motivation.

**Interviewer**: **Mmh, Well, what will be the benefits for the clinic if this method will be applied?**

**Participant**: It will attract… it will attract people to come, because they will then kow that if they attend methadone clinic, we shall be given transport fair.

**Interviewer: Mmh…,**

**Participant:** It will also help to get money for food because many of them do not have the specific source of …so even food will be a problem for them.

**Interviewer: Mmh well, but also, I would like to know what will be the difficulties in providing motivation to methadone users in this clinic, let us say we have succeeded to get the motivation or assistance and our patients are being given motivation which difficulties do you suspect that might happen to methadone users?**

**Participant:** The difficulties which will happen, you know, when you start something you need to have a number; therefore, you can say may be today we have got 200 users, but if you will put a motivation, 400 will come, therefore your budget will be in trouble, that is, may be if we can conduct a survey to know all users who are ready to start using methadone**.**

**Interviewer: Mmh well**

**Participant:** This is the difficulty which I see

**Interviewer: Well, I understand you that, sometimes you will see that the budget will not be enough because it will attract many people.**

**Participant:** Mmmh

**Interviewer: But in the implementation of motivation to methadone users what challenges or obstacles do you expect from the MZRH administration, which challenges will come from administration to implement this issue of motivation?**

**Participant:** The first thing which I see, because that will attract, therefore even the budget might be big

**Interviewer: it can be big Mmh, another thing?**

**Participant:** The second thing will be in providing those motivation.

**Interviewer: They way, how to give them**

**Participant**: That is how do we give them, do we hand them, do we give them through phones, because others do not have phones, or do we put in the bank accounts others do not have bank accounts that means how will be the procedure of providing it.

**Interviewer**: **Well, in the implementation of motivation to methadone users for the aim of modifying behavior what challenges or obstacles do you expect from the users of methadone services?**

**Participant:** From the users?

**Interviewer: Mmh**

**Participant**: From users…… what will happen ……..because I see the methadone users are affected behaviorally, mentally and psychologically, what I see, you can put the motivation let us say we will give 5000 per day, they may start complaining by saying that ooh no!, we are supposed to be given 10,000 they are taking our money!.

**Interviewer: Mmh, so here you are talking about the trust to patient themselves.**

**Participant:** Yes, their trust

**Interviewer: That is, because we are being given this small amount there must be another big amount there, but we are given only this small amount.**

**Participant**: Eeeh, this is why they give us this small amount.

**Interviewer:** **Another thing Mh?**

**Participant**: Another thing… which I see mmmh… we cannot say security, that is to say the one who is providing the motivation at the time of giving that means…

**Interviewer: That means it can lead to violence**

**Participant**: it can cause violence, that is even those who are not…. (**Interviewer:** **who are not supposed to be given)** supposed to be given motivation may come. It can lead to violence and even those who do not deserve it.

**Interviewer: Mmh,**

**Participant**: …and the third thing… is this motivation to be given to all people who attend methadone or there are groups?, that is, there are specific groups let us say we give the motivation to patients, pregnant women, physical disabilities… which system will be applied in giving that motivation?

**Interviewer**: **Mmh Yes, that can be a challenge**

**Interviewer:** **But in the administration of the implementation of this intervention what kind of challenges or obstacles do you expect to come from the methadone providers that is, those who provide services, what challenges may come from them if we put this motivation issue?**

**Participant:** From them?

**Interviewer: Mmh,**

**Participant**: Mmmmh…, service providers… Eeh!, I do know if……if the service providers will be given that motivation or it will be the methadone users only, so we will go back to the innocence or…

**Interviewer: Mmh, that is, if he /she is not getting and is the one who is providing the money, he may desire.**

**Interviewer: Mmh, another thing?**

**Participant:** Another thing from service providers; another thing as I have said, in the groups of providing motivations, is it that not others who are not intended may come?

**Interviewer: Mmh yes, so the challenge will be to know the targeted.**

**Participant:** Mmh,

**Interviewer: Well, Motivation to methadone users. This intervention can be implemented by two types of motivation, there is money motivation and item motivation, which one do you think is much better to be used if we start providing motivation in this clinic?**

**Participant:** Mmh!

**Interviewer: Between money and items**

**Participant:** What I see in motivation, still I believe in putting groups, you can give an item to a person let us say you say that, may be because this one is in need of food; then you decide may be in every Friday you give for instance, grains, milk, or vegetables, but he/she may decide to go and sell, this is what I see, so in this motivation I think let us just give them money.

**Interviewer: Money?**

**Participant:** Eeh,

**Interviewer: Why do you think money?**

**Participant:** Because they need money for transport and food

**Interviewer: Transport fare and food**

**Participant:** I see these are big things that bother them

**Interviewer: They bother them, Mmh, Well.**

**Interviewer: Thank you for your time I have completed my questions, Thank you very much**

**Participant:** Thank you

**End of Interview**

**PARTICIPANT TEN (47 YEARS-------- Z0000036 VLC media player) IF5**

**Interviewer:** If you have any question concerning the study as a participant you can ask the principal investigator and head of continuous education Mbeya Zonal Referral Hospital P.O. Box 419 Mbeya.

**Participant:** Thanks a lot

**Interviewer:** So, you are ready ehee,

**Participant:** I am ready

**Interviewer:** **Well, my direct question to you first, I would like to know your age**

**Participant:** I am 47

**Interviewer: Your marital status?**

**Participant:** I am married

**Interviewer: Who is living with you at home?**

**Participant:** I am living with a husband, but by the time being I am working here, so I am staying alone.

**Interviewer:** **What is your highest level of education?**

**Participant:** Masters

**Interviewer: How many years did you spend to attain your highest level of education?**

**Participant:** It is like 26 years.

**Interviewer: And your source of income?**

**Participant:** I am doing business, and I am a government employee

**Interviewer:** **Well, and for how long you have been a leader at this hospital up to the moment?**

**Participant:** Two years

**Interviewer:** Two years, thank you.

**Interviewer:** **Name any challenges or obstacles that you know as a hospital administrator in methadone services in this hospital**

**Participant:** Aaah… services related to methadone is that, there are youths who come here to be given medicine, but for me also as a leader I see the complaints for those who come to take the medicine, including paying those who escort the medicine from Dar es salaam to Mbeya.

**Interviewer: Ok, fine. Now we will go direct to our questionnaire as it is explaining, the obstacles of implementing motivation to methadone users. I will read to you a little bit of it, and after that, I will ask you questions. Motivation to methadone users is the intervention of behaviour, the directive of this intervention suggests that behaviour change to be more positive when there is a positive motivation, and behaviour change to be more negative when there is a negative motivation. The interventions involve the provision of congratulations and punishment targeting at the occurrence of the expected response. Motivation to methadone users has a great evidence of effectiveness in modifying various behaviours of patients. Now I will ask you questions after those explanations. Tell me what do you understand about motivation to methadone users as part of modifying behaviour?**

**Participant:** Motivation increases effectiveness, whereas it brings about self-awareness to the characters, especially the methadone users, it increases the self -esteem of a character and reduces negative behaviours in the community.

**Interviewer: Ok, thank you, according to your views, why this intervention is not being practiced in methadone clinics of Tanzania?**

**Participant:** Aaah, according to my views, I think it is due the limited funds whereas the needs are high when compared to the available money resources, and also the hospital has a big number of methadone users and therefore, this can make that motivation to be not enough for those who will need it because they are many.

**Interviewer: OK, fine, and what do you think about this intervention, how useful will it be at the methadone clinic?**

**Participant:** If this method will be applied, it will increase responsibility to the user, and he/she will realize that he/she is supposed to do what he is supposed to do including to increase productivity, the reduction of negative events, evil acts and violence in the community.

**Interviewer: Ok, thanks**.

**Interviewer:** …**and what do you think, if we will start these interventions of providing motivation, what kind of difficulties will occur in implementing the motivation to methadone users in this clinic?**

**Participant:** In this clinic, I think it will increase human resources, because if they will know that there is money the number will increase, the human resource in this section will need to be big, and as you know that motivation should also be given to workers who are providing services to them.

**Interviewer: Mmh well.**

**Interviewer: …and in the implementation of motivation to methadone uses what challenges or obstacles do you expect to come from the hospital administration?**

**Participant:** The challenge from the hospital administration will be there if they will not be educated, especially about motivation to methadone users. Therefore, education is important to MZRH administration.

**Interviewer:** **OK well.**

**Interviewer: And in the implementation of motivation to methadone users for the aim of modifying behaviour, what challenges or obstacles do you expect to come from the users of methadone services?**

**Participant:** The challenge is to have a big number of users, the number of users I believe it will increase because they will know that there is motivation, but also another challenge is for servants whereas human resource will be no longer enough, another challenge will be to provide education in other words we say customer care, because sometimes when patients are many it is likely that people will get tired and may use a language which is not appropriate, another challenge is to provide education to service providers so as to provide services to clients and use appropriate language.

**Interviewer: Ok, well, and in the administration of the implementation of this intervention what challenges or obstacles do you expect to come from methadone service providers?**

**Participant:** The challenges will be for the services providers to claim for extra time money including other rights in accordance with the procedures, regulations, as stipulated in the public service in addition to its guidelines.

**Interviewer:** **Ok, thank you madam for your participation, but I would like to ask you the last question that according to your views that, when we start to provide motivation what do you suggest to be the motivation money or items?**

**Participant:** It should be both of them

**Interviewer: Both of them, and what do you think to be the reason for providing both of them?**

**Participant:** The reason is that, for example if you give a person an item you will find that another user has no clothes, has no what, so if he will be given it might help him/her because if you give him/her money to buy clothes he /she will not buy, but the reason to provide money is that another person is coming from home on foot he/she has no transport fair, no food so if you give him/her money will help him as bus fair or food this is why I said we should provide both of them.

**Interviewer: Thank you madam for your cooperation, have good day**

**Participant:** Thank you too, you are welcome.

**End**

**PARTICIPANT NO. NINE (39 YEARS-------- Z0000035 VLC media player) IF6**

Interviewer: are ready to continue to participate in this study?

Participant: Yes, I am ready

Interviewer: Ok, thank you, now, I will read to you the questionnaire on the obstacles in the implementation of motivation to methadone users and service providers

**Interviewer:** **I would like to know your age**

**Participant:** I am 39

**Participant:** **Your marital status?**

**Interviewer:** I am married

**Interviewer:** **whom are living with?**

**Participant:** I live with a husband, children, and the house girl.

**Interviewer**: **What is your religion?**

**Participant:** Muslim

**Interviewer:** **What is your highest level of education?**

**Participant:** Form four

**Interviewer:** **What was the duration spent in studying your highest level of education?**

**Participant:** Three years

**Interviewer: What is your source of income?**

**Participant:** Employment

**Interviewer: For how long you have been a leader in this hospital?**

**Interviewer: I mean being the head of department**

**Participant:** Four years

**Interviewer: Name the difficulties and challenges related to methadone services that you know.**

**Participant:** The difficulties that we face is the difficulties in understanding of clients, many clients do not understand,

**Interviewer:** OK, they do not understand.

**Interviewer:** **Well, I will read to you the obstacles of implementing motivation to methadone users, a few explanations, then I will ask you questions. Motivation to methadone users is the intervention of behaviour. The system of this intervention suggests that, behaviour changes to be more positive when there is positive intervention and change to be more negative if they are provided with negative motivation. The implementation involves the provision of congratulations and punishment aiming at the occurrence of the expected outcome. Motivation to people who use methadone has a great evidence in of effectiveness in changing various behaviours of patients. Now I would like to ask you these questions, tell me whatever you understand about the provision of motivation as part of changing behaviour.**

**Participant:** (silence)

**Interviewer: That means when they say motivation to patients what do you understand?**

**Participant:** I think my understanding of motivation is for example if you give them money because many methadone patients are in need of residence services, money, and need to be close to their families.

**Interviewer:** **The closeness with their families, fine.**

**Interviewer: In your views why do you think this method of providing motivation to methadone patients is not applied in Tanzania?**

**Participant:** I think our system was not well arranged right from the beginning.

**Interviewer*:* What do you think will be the benefits to methadone clinic in case these procedures of providing motivation will start to be applied?**

**Participant:** They will know that there is another social service provided to them apart from medicine.

**Interviewer**: **Ok, and what do you think, if we start providing this service to patients what challenges do you think will happen?**

**Participant:** Mmm… challenges… I think the challenges will be there for those who will not be getting incentives, they will be want to get it and they might not know why we are giving others but not them, even if we will explain to them, they will not easily acknowledge ,rather they will believe that we are favoring them.

**Interviewer: OK fine, in the implementation of motivation to methadone users what challenges or obstacles do you think will come from the hospital administration?**

**Participant:** Can you repeat the question?

**Interviewer:** **That means, when we start to provide these motivations, the hospital must be in involved, may be the hospital will be involved in providing those things. Now what do you think will be the challenges that we might get from the hospital administration in providing those motivations?**

**Participant:** The challenge is delays.

**Interviewer:** **Delays, fine.**

**Interviewer:** **In the implementation of motivation to methadone users what do you think will be the obstacles that will happen or come form the service providers who provide services to methadone patients?**

**Participant:** Mmh, challenges… Also, I think the service providers should be getting something

**Interviewer: That means if they will not be getting anything it is a challenge.**

**Participant:** Yes

**Interviewer:** **OK, motivation to methadone users; in implementing it there are two types of motivation; the money motivation and motivation by giving things, which would you prefer or suggest between them in case we decide to implement this issue?**

**Participant:** Motivation of money, because things can be given to a person which he/she has them already, but if you give money, one will know what he does not have and look for it

**Interviewer:** **Mmh, fine, OK. Thank you for your cooperation. Thank you, we have completed all the questions in the questionnaire. Thank you very much.**

**Participant:** OK, Thanks

**End**

**PARTICIPANT NO. SIX (22 YEARS----- Z0000025-VLC media player) IM5**

**Interviewer:** After reading the consent form to you, are you ready to continue to participate in this study?

**Participant**: Yes, I am ready

**Interviewer: Ok, you are warmly welcome I will continue asking you questions. I would like to know your age**

**Participant**: I am 22 years

**Interviewer: (Twenty-two years) Your marital status?**

**Participant:** Not married

**Interviewer: (Not married), Who are you living with?**

**Participant:** I am staying with parents

**Investigator: (with parents) … What is your religion?**

**Participant:** Christian

**Investigator: (Christian)… What is your highest level of education?**

**Participant:** Certificate level

**Investigator: (Certificate level) …How many years did you spend to study in this level of your education?**

**Participant:** One year

**Investigator: (One year) What is your source of income?**

**Participant:** Business

**Interviewer: … If you are employed name the job that makes you get salary**

**Participant…**I am not employed

**Interviewer:** You’re not employed

**Participant:** Yes

**Interviewer: Well, no problem, for how long have you been providing methadone services here to date?**

**Participant:** This is a third Month

**Interviewer**: **(Third Month), mention the difficulties and challenges which you encounter in relation to methadone services.**

**Participant…….** the big challenge is for these methadone users **(Interviewer: Mmh**) I can say that they do not follow the preset instructions, (**interviewer**: they do not follow the rules, mmh) such as how they put on attire **(Interviewer**: Mmh**) time** to arrive, they are instructed that the end of service is this time, but they come late

**Interviewer:** (reciting: they come late)

**Interviewer: Perfect, now I will read to you, the obstacles of implementing motivation to methadone users. I will read to you so that you can understand a little bit, then I will ask you some questions below which is the major aim of our study. Motivation to methadone users is the intervention of behavior, the procedures of this intervention suggests that, behavior change to be more positive when there is a positive motivation and declines to be more negative if they will be provided with negative motivation. The implementation involves the system of providing congratulation or punishment with a focus on the occurrence of the expected response. Motivation to methadone users contains substantial evidence in the modification of different behaviors of patients. Now I want you to tell me what you understand by motivation to methadone users as part of behavior modification?**

**Participant:** May be…. what I understand is that, if they will be given those…. any motivation, it will make them change to a greater extend.

**Interviewer: Aaah that means, you believe that motivation will bring about changes**

**Participant:** Yes

**Interviewer: Mmh, but also according to your views why this intervention is not being used in methadone clinics in Tanzania. Why do you think we are not using these methods?**

**Participant………**(silence)

**Interviewer: It means, what do you guess to be the reasons for not using this method to provide motivation to methadone patients?**

**Participant:** May be because of themselves, the way they bring difficulties in working

**Interviewer: ( Mmh difficulties in working), Another thing?**

**Participant……**…(silence)

**Interviewer: any other thing that you think as to why Tanzanian clinics do not use this method of giving motivation?**

**Participant**: I think may be because of methadone users themselves do not have a good cooperation with workers

**Interviewer: Mmh cooperation**

**Interviewer: Well, what do you think if the motivation comes, that is now the motivation is here and patients are being given; such as a person who came early at the clinic is given some money? What do you think will be the usefulness to the clinic if we will start giving motivation?**

**Participant:** it will change behaviors to some people

**Interviewer: Mmh, another thing? Another thing which will be helpful at the clinic?**

**Participant:** It will pronounce the good reputation of the relevant clinic

**Interviewer:** **Reputation of clinic, another thing?**

**Participant**: …(silence)

**Interviewer: OK, if we will start providing these motivations what do you think will be the difficulties in implementing this motivation to methadone users in this clinic, what difficulties we will get in providing motivation to patients in this clinic?**

**Participant:** The difficulties will be there if methadone users will not show cooperation

**Interviewer: Mmh not showing cooperation, another thing?**

**Participant:** I think it is only that.

**Interviewer: Mmh**

**Interviewer: Again, in the implementation of motivation to methadone users what are the challenges or obstacles do you expect to come from the hospital leaders, that is, now we are supposed to start providing motivation what do you think will be the challenges from the hospital administration?**

**Participant:** Follow up

**Interviewer: Follow up, another thing?**

**Participant:** Only that

**Interviewer: Only that**

**Interviewer: In the implementation of the provision of motivation to methadone users for the aim of modifying their behavior, what are the challenges or obstacles we expect to get from the user of methadone services, that is now the patients who use methadone, which challenges do we expect to get from them if these motivations will come to be given when they do well?**

**Participant:** …the challenge which I see is that of personal hatred for those who will not be in good understanding.

**Interviewer: ahaa, personal hatred, another thing?**

**Participant…** (silence)

**Interviewer: Another thing that you see is a challenge that will come from patients?**

**Participant**: …Mmm!...(Silence)

**Interviewer: None eeh, Ok**

**Interviewer**: **In the implementation of this intervention what challenges or obstacles do you expect to come from the methadone service providers, that is what challenges may come from them?**

**Participant:** To provide education to the users

**Interviewer: Mmm, to provide education, another thing?**

**Participant…….** (silence)

**Interviewer: Only those**

**Participant:** Yes

**Interviewer: OK well, but also this interventions can be provided by using two types of motivation, you can give patients money or stuffs, let us say those who will come early we will give them sugar or may be those who are not taking other substance to mix with methadone we will giving them money, now in this section, you as a methadone worker what do you suggest money or stuffs?**

**Participant:** stuffs

**Interviewer: Mmh, now, why do you see stuffs to be good for our patients to be given?**

**Participant:** They will bring about development for them rather than giving them money

**Interviewer: Mmh, development**

**Interviewer: Thank you our questions end here, thank you for your cooperation. Thank you very much.**

**Participant:** Thank you

**Interviewer: Thanks**

**End**

**GROUP INTERVIEW DATA SETS**

**Interviewer**: Now we shall start with our questionnaire and I will be recording, the first section says, please explain what do you understand in relation to barriers in the implementation of motivation to methadone users, likewise, estimate your understanding in which you can mention the benefits of the service to methadone users, here it wants us to know about the obstacles, as we said before that; what obstacles which make us not to be able to provide motivation. So, what do you understand about the obstacles of implementation of motivation in methadone services?

**Participant**: Silence

**Interviewer:** That is, whatever you know about the obstacles of providing motivation to methadone users.

**Participant 1**: … If we start maybe by this implementation of motivation to methadone users, I think first the responsible centre to have enough strategies for that, and when we are talking about motivation, we are talking directly about capital.

**Interviewer**: Capital yes,

**Participant 1**: You see, so you need to have enough capital to fulfill this.

I**nterviewer**: Mm

**Participant 1:** To fulfill motivation to methadone users.

**Interviewer:** OK, anybody else here, who have something in understanding motivation?

**Participant 2**: For me I think another thing which is big it is not a centre as a centre

**Interviewer**: Mm

**Participant 2:** …. But even the government started things like these, but they did not put into serious consideration things like these, that is, what things will simplify in the provision of big motivation, for the users of those clinics.

**Interviewer**: Mm

**Participant 2**: Therefore, the government also was supposed to consider and allocate a certain budget which can enhance it, because a centre as a centre has no generation funds for that, for example even when you try to see even the medicine is a burden to the centre and now if you add the motivation issues.

**Interviewer**: Issues of motivation

**Participant 2:** Therefore, the big issue here, the government is the target.

**Interviewer:** To the government Mm

**Participant 2:** Yes, it is supposed to have an allocated budget.

**Interviewer**: Mm OK, Well.

**Participant 3:** I want also to contribute at this part that; despite the government, in our community also, there are several institutions especially in the respective regions and not necessarily to be central, we have the sponsors who are within the region, it is better also to explain to those people about something like that. We can gather them at any point even at the institution level, we can gather them privately, then they can be explained about the issue, there are several organizations like; Pepsi, Vodacom, Minerals…, we can explain to them, and not only looking at the government alone, while we have other stakeholders

**Interviewer**: We have others

**Participant 3**: Eeeh, the institution should look for other ways on how to talk with those people, even the government itself, but in general we as a region we might have such a strategy also to see the institutions and tell them the importance and disadvantages of these problems, I think they can devote.

**Interviewer:** They can devote

**Participant 3:** Mm,

**Interviewer:** OK, but also at this same part it says, approximate your understanding, can you name the advantages of the service to methadone users that is, what do you think will be the advantages of motivation for methadone users, that means if motivation will be provided at the methadone clinic, what will be the benefits?

**Participant 4:** First, our client will behave well, when we see that they have behaviours which are not accepted, if it will be used as a motivation that whoever will come wearing sanders will not be given the motivation which is given to others, the following day all of them will come in the acceptable shoes. So, it will increase…it will increase…discipline.

**Interviewer**: behaviour modification

**Participant** 4: Yeah.

**Interviewer:** That is, to follow the regulations

**Participant 4:** Mm

**Interviewer:** Other benefits?

**Participant 1:** To add there, another benefit that can be gained is, as we continue to provide motivation, the number of patients form the community will increase as they will come to know that they recover and at the same time they are provided with motivation, so it will be a double benefit for them.

**Interviewer:** So, the number will increase.

**Participant 1:** It will increase

I**nterviewer**: Mm

**Participant 5**: I think the way how my brother has said, even us we were trying to deceive them in the streets, this is why others have stopped, because we told them that after a few days you will get something, and when they came, they found that nothing is being provided and when they ask, we tell them that they should recover first because “you did not come here for money but for treatment”, others came back and said aah!, let us not follow money but the treatment.

**Interviewer:** OK, thank you, we will go in the second section, it has three questions, the first one says; why do you think this service is not famous or not applied in Tanzania?

**Participant 4**: This motivation services?

**Interviewer**: Motivation

**Participant 3:** Here I think the issue which comes here, gives the answer of financial issue.

**Interviewer:** Financial, yes.

**Participant 3:** Eeeh, and then, even if when you compare our expenditure with our fellow countries, there is a big difference, when we look at our expenditure ratio it is very small when compared to our fellow countries. So, they have done so to get rid of them, they have done so depending on their big number of the population of users when compared to us. Therefore, this can be one of the reasons.

**Interviewer:** One of the reasons

**Participant 3:** Yes

**Interviewer:** That is, we have a few users, hence priority cannot be given there.

**Participant 3:** Yes

**Interviewer:** Another thing that make us not to use it while we understand it can help in behaviour modification?

**Participant 2:** According to my views, we are not using this because we are relying too much on dependence, we think that it should be the government to do so, but even ourselves as civil servants we can provide motivation to these people because it is not something huge.

**Interviewer:** Mh, mmh, Okay, I am moving to the second question, can you tell me what is the good way of implementing this one, for example in case we have decided to implement this method, which method is the best in providing this motivation?

**Participant 1**… There…what I think, may be the best method and which is safe it is not to make people use this thing for a prolonged time, either when a person has a good consistence let him/her be graduated, it can be helping the person also to be motivated.

**Interviewer:** Mm, in motivation, fine, another thing?

**Participant 4:** and also, I want to add there, by saying (laughs) maybe the day a person is graduating we can help him/her to open a business.

**Interviewer**: A capital

**Participant 4:** We can provide a good guidance to a person to go and do something else outside there, that will make him not to go back to bad groups and will make him to be busy with business or work. The motivation might be of that design. I think even a person who is starting using methadone treatment will be thinking that let me take medication well, let me adhere to regulations so that I can graduate and be helped in that way as well.

**Interviewer:** OK, it is a good point. I will go to the third, what is your opinion on the institutions which provide motivation to methadone users, what should be done in order to provide motivation, what should be done in implementing? We are basing on implementation.

**Participant 3:** In my side what I can see, first let us look at that motivation

**Interviewer:** Mmh,

**Participant 3:** Let us not base too much on something relating to money, money, motivation is of many kinds.

**Interviewer:** Mmmh,

**Participant 3:** Therefore, as a centre we can arrange a system that those who attend methadone clinic to learn a certain skill, that is motivation, because others will come just by thinking that let me join the methadone clinic to get methadone treatment including a certain skill, because I know I will get a certain skill, therefore by learning the skills he will distribute the skills to others and it will facilitate even to get other people to attend to the methadone clinic and make a fast change, because they will know that if I miss the attendance to the clinic I will not get the skills, therefore the skills should be of a wide variations because the skills can educate people of good gardening etc., and when considering that Mbeya is a good area for things like these.

**Interviewer:** Mh, fine, I have understood you.

**Participant 2:** Let us go back to the leader there, who earlier said that we can also involve private companies, the act of involving them is not just involving on the issue of money or what but to involve them by telling them that we have youths who use methadone; how are we going to help them after graduation, we may bring them to you such as to Pepsi company so that they can work in activities such as cleaning or whatever, it can also be a good thing.

**Interviewer:** It can be good thing, Okay, fine, but also, I am moving to another section which says; what value costs is suitable, or what do you suggest for implementation of motivation to methadone users, if we say costs it is as one of us here who said those who will come early, we will provide a certain amount may be money for transport, or a certain thing, in which form we should provide. For example, somebody has done well and we give maize flour in which amount? 20kg?, several kg? Here it shows costs in things and money. For example, we have decided to make things how much they should cost?

**Participant 2:** My idea about the costs, we should not put the flat rates, but we shall look for the needs of a specific person, instead of saying that may be all who graduate we will give sugar or maize flour or whatever, but we should look at the needs of the person we are giving and his/her environment, eeh, that means we can help him/her for something which is of short time like maize flour he will eat and finish and then? Maybe it was only 5 kilograms, so we need to consider the need of person who is graduating, but also to look for something that can be of benefits to the person. However, putting the exact amount as you say can be right because you cannot fulfill all of the person’s needs for the whole Month as it will be a big amount of money, I remember one day one of them said that “I do not have money for only processing maize at the grinding machine” I do not know if he was deceiving me or what, but he talked with a real feeling.

**Interviewer**: Mh, He had maize, but did not have money for grinding

**Participant 2:** Eeeh! In that day he had a big need, the need may reach fifty thousand but this is his/her need.

**Interviewer:** Eeh, anybody about cost issues? But also, we are looking even to those who had done well. Maybe we have tested them and their urines have no substances that is, if this happens then, how they should be motivated?

**Participant 2:** To encourage them.

**Interviewer:** Mmh, so that they continue with good behaviours so what should be the costs?

**Participant 2:** For me the amount of money for a person who has tested negative in order to support him/her and encourage even if you give 5000sh. Is a big amount of money.

**Interviewer:** Is a big money

**Participant 2:** Eeeh, yes, 5000 shillings to get it for free without doing anything, is a bonus, he will do his things with that amount of money.

**Interviewer:** He may use the money for transport for the whole week.

**Interviewer:** Well, now we are going to the policy and administration. What are the policies and administration issues which are supposed to be prioritized in order to succeed in the provision of motivation to the users of methadone services?

**Participants:** (silence)

**Interviewer:** Issues of policy and administration, here what are the issues perhaps the leaders of the hospital, national leaders what type of issues maybe, can be done in policy and administration that can help the methadone services be successful?

**Participant 3:** First of all, what I see in my opinions in making policies, there must be a day, a special day to say that this is a day for substances users, and not only having the special day, but also the leaders of higher positions should attend in those vacations, because this will make a person to ask himself/herself after seeing leaders of higher rank positions have attended, that is; I see! even the president, or a member of the parliament or a minister has attended in this day, so it is something that will remain in person’s mindset that this day has been given a valuable attention by the country, but when it is quite no special day, no what, they should look carefully at this matter.

**Interviewer**: So, you think the “Substance of Abuse Day” will bring about motivation and more understanding among people.

**Participant 3:** Understanding, because there are people even though now we are talking about the drugs of abuse, others do not know, and others do use without knowing that these are substances of abuse, and it is true that if you trace them those who used, they did not plan that this time, I want to start using substance, no, it was just a mob psychology to others, so it is to instill community awareness.

**Interviewer:** Awareness to the community

**Participant 1:** But to add on that, he has talked about awareness, but another thing I think we mostly miss is promoting this thing, for example there are things which are given priorities such as family planning, so you can hear even on a radio, therefore, even this one, they can make a certain kind of a promotion like in radio and television.

**Interviewer:** Mmh.

**Participant 2:** I have an idea also in that, it should not be to the users alone, but even to those who distribute the substances, because the routes of distribution are there and the government……*.*…. I was thinking about the authority too, to impose punishment, we have seen vividly that they make laws, they fight against it, but the punishment is not as big as that of the rape which is straight forward. A person who distributes drugs will be caught, but he will not be punished because of the power of money, I do not know if they give bribes or what, whatever, but the law instruments should impose heavy laws for those who distribute those things or those that make that system to be their way of generating income for them, because it is them who provide us with the community which has suffered to this extend. There must be a strict law.

**Interviewer:** There must be a strict law, fine, now we will get in the last section of “others”. We say that, in this discussion we have made, maybe there are questions in relation to motivation to people who use methadone services that is the questions which we have here at this centre, do you have any question?

**Participant 3:** I have a little explanation; it is not a question or what.

**Interviewer:** No problem

**Participant 3:** It is just to say…. should not recognize the users only but also it should recognize the service providers, because most often the environment of the service provider is also very difficult, so, if it is the government or community, should recognize those people on the numerous services they provide, because you will see in other areas, those who provide this service have been given this kind of priority.

**Interviewer**: This priority mmh.

**Participant 3:** Eeh, so that, they should not feel as if they are discriminated, because if the service provider will be discriminated, I do not think that he/she will remember the one who receives that service.

**Interviewer:** The receiver of the service will be forgotten

**Participant 3:** Eeh, so those are the things to consider

**Interviewer**: Eeh, I think it is a good thing.

**Participant 2:** I also ask for we service providers to change our attitude towards the community, instead of contributing a lot of money for things like wedding, we can put things like these, we can contribute to things like these as we do to things like wedding, because you will see that we are looking for motivation may be to a 300 hundred people we have, if we had 15 million or 30 million here, they can push a lot of things, here is the same amount as collected in a wedding

**Interviewer:** Yeah, even for some Months

**Participant 2:** Yeah, we give pleasure to ourselves, we find that it is better to go and eat instead of giving to help the community

**Interviewer:** Mmh, anyone who wants to contribute?

**Participant 1**: At the same point in addition is that, every institution which provide services, it should provide assistance, it should provide a fund, maybe to have a fundraising at the specific centre to deal with this issue.

**Interviewer**: Fundraising, mmh.

**Participant 2:** Do you know why we are saying this? We, for example at the Regional hospital the corridors were built by the workers, we started, each worker contributed 100.000Tsh, and we invited people and we contributed again in the second phase and people entered, hey! We came to know that now we can start to do things on our own.

**Interviewer:** We can start and others can join

**Participant 2:** Yes, they can join us, and when people see that you have started, they cannot leave it.

**Interviewer:** Mmh, when we see other departments are doing it and therefore even us in motivation, we can start on our own

**Participant 2:** Yes

**Interviewer:** Is there any other opinion?

**Participants:** Silence

**Interviewer:** Let me thank you all for participating. Thank you very much.

All participants: Thanks
