# Supplementary material for: Barriers to implementing contingency management at a methadone treatment clinic: A qualitative study at a tertiary hospital in Tanzania
Source: PLoS One. 2025 Mar 3;20(3):e0314168. doi: 10.1371/journal.pone.0314168 (PMC11875383; doi:10.1371/journal.pone.0314168)
Supplement: Attachment — Submitted filename: Response to Reviewers.docx [file pone.0314168.s005.docx]

Mirembe National Mental Health Hospital, P.O. Box 910, Dodoma, Tanzania

Email: alodala12@@gmail.com

31/03/2024

**The editor,**

**PLOS ONE Journal**

Please find enclosed manuscript: **Barriers to** **implementing Contingency management at a methadone treatment clinic. A qualitative study at a tertiary Hospital in Tanzania;** which I submitted to be considered for publication in the journal.

I thank you and your team to accept and forward it for reviews. I acknowledge to receive reviewer’s comments which I have been working on since January 3^rd^ ,2024

Thank you for accepting request for extension of time to work on the reviewer’s comments.

I have attended item by item the reviewer’s comments and now re-submitting according the directions indicated in your e mail.

Thank you.

I trust your team,

Kind regards,

Dr. Paul S. Lawala

Response to review comments

| S/N | Comment | Response |
| --- | --- | --- |
|  | Reviewer #1 |  |
| 1. | This manuscript explores barriers to implementing contingency management in a hospital methadone treatment program in Tanzania. This is a generally well-written paper and a very important topic in light of the global opioid crisis. While most comments are minor, one major limitation may be the lack of opinions from methadone treatment participant clients as well as the inclusion of one treatment client in the focus group among program staff and providers. This study would have been much stronger and more valid if either the clients were not included at all or if more clients were included and were offered participation in their own focus group. | Thank you for your complement. The client included in the focus group discussion was not a regular client rather an overall leader of the group and therefore it deemed suitable for us to include this client in a focus group discussion with health care providers as the client was conversant with the hospital administration and hospital operations. While we believe that our study generated important information on barriers on contingency management, we acknowledge a limitation that if a separate FGD on clients was conducted perhaps there could be additional information. |
| 2. | **Abstract**  Does “methadone user” means individuals who are being treated at the methadone clinic? This should probably be “methadone treatment client” or something similar. Methadone user seems like someone who uses methadone illicitly. | Thank you for your question. Yes, “methadone user” means individuals who are being treated at the methadone clinic. We accept your suggestion “methadone treatment client” has been used instead of methadone user in the revised manuscript. |
| 3. | **Background**  Maybe replace “mental” with “behavioral” in “mental health disorders” (first paragraph) as it is a bit less stigmatizing. | Thank you for your observation  The term mental and behavioral disorders is used in international classification guidelines, for example in ICD10. Therefore, we have changed from metal health disorders to mental and behavioral disorders |
| 4. | Please update some of the refs in paragraph one. For instance, refs 1, 5, and 6 are outdated | Thank you for your comment. We have updated the references as reflected in the revised manuscript |
| 5. | Line 59, second paragraph: Contingency management is recommended by who? | Thank you for this observation! There was a missing word at the end of this sentence, it has been corrected and reads, “recommended by previous research as an adjuvant treatment for substance use |
| 6. | The introduction needs a clearer discussion of how contingency management is used in opioid treatment (second paragraph of background). Please add from the literature what behaviors may be reinforced through CM as well as examples of the incentives. For instance, monetary, gift cards, and prizes may be offered, but there is very little mention of that in the manuscripts. This would help support the financial barrier finding cited in the manuscript. Also please add some examples of outcomes that have improved from the use of contingency management in methadone treatment from the literature. Some more recent studies would also be important to add. | Thank for your suggestion. We have addressed this in the revised manuscript citing also previous research on the topic |
| 7. | Also in paragraph 2, the citation 7 (“Implementation support for contingency management: preferences of opioid treatment program leaders and staff” may not be the most appropriate reference to support the statement that CM improves substance use outcomes, as this is a qualitative study exploring OTP staff preferences. | Thank for your suggestion. We have addressed this concern in the revised manuscript and suitable citation inserted |
| 8. | First line of second paragraph: Instead of saying no references exist, it may be better to say that the literature is sparse or something similar. | Thank you for your comment  We have replaced the sentence “No literature exists” to literature regarding information on the use of contingency management is scanty, specifically in methadone clinics |
| 9. | **Methods**  Were participants paid for study participation? Also please mention if consent was obtained | Thank you for your question.  No, participants were not paid to participate in this study.  All participants signed a written informed consent form. |
| 10. | Were participants from the focus group different than the participants completing IDIs? | Yes, participants in the focus group discussion were different from those who completed IDIs. While we selected IDIs participants purposively by virtue of their knowledge and experience on the matter in order to get rich information, we selected a different group of participants to participate in FGDs |
| 11. | Why was one methadone treatment client included in the focus group with the providers? If there were not enough treatment clients to participate in the focus group, that client probably should not have been included. First, the power structure where a patient from a stigmatized group (substance user) sits in the same focus group as hospital providers (who may be directly or indirectly responsible for treating these patients) may create a situation where the client or providers may not feel as though they can provide honest feedback (as mentioned in the manuscript, there are trust issues). Additionally, questions asked of patients regarding certain interventions that may be implemented in a program would be very different than those asked of staff and providers. Having one client in with a group of providers may contaminate feedback on provider/administration barriers. This should be included as a limitation. | Thank you for this question. There were enough methadone clients. But the client included in the focus group discussion was not a regular client rather an overall leader of the group and therefore it deemed suitable for us to include this client in a focus group discussion with health care providers as the client was conversant with the hospital administration and hospital operations. However, we have also included it as one of the limitations thus calling for further studies that could exclusively interview clients |
| 12. | More information should be added on how interview guides were developed. Related to comment 3, above, where questions different for staff and clients? | Thank you for this question. Interview guides were developed guided by our conceptual framework , the CFIR “ with specific reference to previous studies on contingency management .Since we aimed at exploring the same information from different participants (triangulating information given by providers and clients), the questions were the same |
| 13. | **Results**  I am not sure that figure 1 adds anything to the manuscript | Thank you for your consideration. Having a pictorial presentation, aids a reader to have a quick spot of the identified barriers |
| 14. | Page 7, line 165: the word “if” may be missing from “in a structured way it could improve…” | The whole sentence revisited and corrected |
| 15. | Page 10, line 248: I believe this should be “latter”, not “ladder” barrier | Yes. The word ladder was mistaken for latter. It is corrected now |
| 16. | There are several pages where “.” (periods) and other punctuation are missing (many examples on page 10). The authors should carefully examine the manuscript and edit where needed. | The missing periods and other missing signs are inserted in the revised document |
| 17. | Page 10, line 242: “fair” should be “fare” (for transport). | Noted! The word fair was mistaken for fare. It is corrected now |
| 18. | The word “motivating” is used throughout (e.g., “motivating” clients). It may be more appropriate to use the word “incentivizing” in most places (e.g., page 10, line 251, “motivating clients is not part of the routine practice.”). | Thank you for this observation. The use of the word “incentivizing” has been applied instead of “motivating” |
| 19. | Page 10, line 253: where it says “reported presented”: it seems like one of these words should be deleted. | Correct  The word presented is deleted |
| 20. | **Discussion**  The authors have written a strong discussion section that includes not only a summary of the barriers found during the study, but also potential solutions from the literature | Thank you for this observation and endorsement |
| 21. | Page 11, line 276: “awareness” maybe should be “lack of awareness”? | Thank you!  This sentence now reads “contingency management has not been implemented widely due to lack of awareness among psychiatrists and mental health professionals” |
| 22. | Page 11, line 278: “motivate” (or similar) may be a better word than “sharpen” | The word “strengthen” is used instead of “sharpen” in the revised document |
| 23. | Page 11, line 281: perhaps change “not using drugs other than methadone” to “not using substances other than prescribed methadone”. | Thank you for your comment, The sentence “Not using drugs other than methadone” replaced with  “Not using substances other than prescribed methadone" |
| 24. | Page 11, lines 283-284: “after target behaviors performed” is written twice. Is this an error? | There was an error, it is corrected in the revised manuscript and reads;  “Consistent with findings from other reports, behavioral controls are suggested through adding the contingency after the target behavior occurs and removing the contingency when the behavior is not performed” |
| 25. | Page 11, line 286: What is meant by “reducing defaulting” in this context? | Defaulting means failure to attend clinics, since all clients are scheduled to attend clinic daily after their official registration with the methadone clinic  “Reducing defaulting” in this context means reducing failures to attend clinics |
| 26. | Page 11, line 289: What is meant by “in monthly or quarterly order”? | “In monthly or quarterly order”? means performing the listed activities scheduled monthly or every three months in a year. |
| 27. | Page 12, line 305: What are the “additional opioids” that are mentioned as medication costs? | “Additional opioids” means pain killers such as morphine or tramadol |
| 28. | Page 12, line 314: that healthcare workers felt they deserved incentives for providing CM is a finding that was not mentioned in the Results section. It should be added there | Thank you , we have added it |
| 29. | Page 13 line 330: The sentence that begins “The role of community approach…” and the next two sentences are unclear/awkward as written. Please revise this section and clarify the information that is being communicated. For example, what are the authors meaning by “community-facility linkage”? | Thank you, revised!  In Tanzania before clients are registered for methadone services at the hospital level, clients are recruited by community-based Organizations. Following recruitment, the clients undergo basic training on methadone and methadone services at the community level. The community organizations then link the clients with the hospital for registration process. This is what we call community facility linkage |
| 30. | Tables  Table 2: It seems as though many of the quotes included in the table are the same quotes from the manuscript. It would be good if the authors could add more new quotes to support the themes. | Thank you for your observation. We have added some new quotes |
| 31. | Similar to comment 1, it is hard to tell how much representation there is among participants whose quotes are listed. For instance, the table is dominated by quotes from 34 y.o. male HCW and 28 y.o. female HCW, as well as some other examples of this. | Thank you for your observation, we have included quotes from diverse participants |
| 32. | Page 24, 3rd quote: transport “fair” should be “fare” | Thanks for your observation  Correction completed. The word “fair” corrected to “fare” |
| 33. | Table 2: The first quote listed under theme 2 (financing) is included under the theme “trust” in the manuscript. | Thank you for your observation , the quote is a financing issue but also a trust issue in the case people perceive that there are no clear grounds for delays in payment . It is therefore reflected both in the financing and trust theme |
| 34. | Page 26, “motivation of health workers” is not listed as a theme in the manuscript. It is listed as “workload” in the MS. | Thank you . We have changed this to workload but expanded the need to incentivize health care workers amid the workload to sustain their motivation |
| 35. | Page 26: I do not see anything in the MS Results about compensating health workers’ time or health workers should be given incentives to implement CM. | Thank you for your observation. We have added in the results section |
| 36. | Page 26, first quote: This quote seems incomplete (ends with “or”). | Thank you for your comment. The or at the end has been removed and the sentence edited to reflect what was said by the particioant  *.* |
| 37. | Page 26, second quote: identifying the quote as being from someone who is an administrator, 45, male may lead to issues with confidentiality/privacy as it may be identifiable. | We have changed the way the quotes are presented to ensure anonymity |
| 38. | Page 26: Under client behaviors, what is “supervising agreed criteria”? Also, “time needed for services”, “motivation for service providers”, and “time constraints” do not seem to fit under this theme and were not discussed in the manuscript. | Thank you for your observations. We have rephrased the wording to reflect client behaviors |
|  | Reviewer # 2 |  |
| 1. | Thank you for your invitation to review this qualitative study that used a CFIR framework to explore the implementation barriers related to contingency management for people who use drugs in a tertiary hospital in Tanzania. The paper identified 6 main themes as barriers to implementing contingency management in the Opioid Treatment Program including 1) awareness and knowledge; 2) Financial barriers to implementation; 3) Trust; 4) Workload; 5) Client behaviors; and 6) Clinic culture  Overall, the manuscript is informative and would potentially add to existing knowledge regarding the barriers to implementing contingency management in other developed countries. However, I think the paper would benefit from language editing (For example, I noticed the authors repeatedly use "substance abuse" instead of "substance use" or places with minor grammar/typographical errors | Thank you for your comments  Language editing is taken as a priority consideration throughout the revised manuscript  The word “substance abuse” will be replaced by substance use in the revised manuscript |
